# Direct visualization of replication and R-loop collision using single-molecule imaging

**DOI:** 10.1093/nar/gkad1101

**Published:** 2023-11-22

**Authors:** Subin Kim, Woo Hee Shin, Yujin Kang, Hongtae Kim, Ja Yil Lee

**Affiliations:** Department of Biological Sciences, Ulsan National Institute of Science and Technology, Ulsan 44919, Republic of Korea; Department of Biological Sciences, Ulsan National Institute of Science and Technology, Ulsan 44919, Republic of Korea; Department of Biological Sciences, Ulsan National Institute of Science and Technology, Ulsan 44919, Republic of Korea; Department of Biological Sciences, Ulsan National Institute of Science and Technology, Ulsan 44919, Republic of Korea; Department of Biological Sciences, Ulsan National Institute of Science and Technology, Ulsan 44919, Republic of Korea; Institute of Basic Science Center for Genomic Integrity, Ulsan 44919, Republic of Korea

## Abstract

R-loops are three-stranded nucleic acid structures that can cause replication stress by blocking replication fork progression. However, the detailed mechanism underlying the collision of DNA replication forks and R-loops remains elusive. To investigate how R-loops induce replication stress, we use single-molecule fluorescence imaging to directly visualize the collision of replicating Phi29 DNA polymerase (Phi29 DNAp), the simplest replication system, and R-loops. We demonstrate that a single R-loop can block replication, and the blockage is more pronounced when an RNA–DNA hybrid is on the non-template strand. We show that this asymmetry results from secondary structure formation on the non-template strand, which impedes the progression of Phi29 DNAp. We also show that G-quadruplex formation on the displaced single-stranded DNA in an R-loop enhances the replication stalling. Moreover, we observe the collision between Phi29 DNAp and RNA transcripts synthesized by T7 RNA polymerase (T7 RNAp). RNA transcripts cause more stalling because of the presence of T7 RNAp. Our work provides insights into how R-loops impede DNA replication at single-molecule resolution.

## Graphical Abstract

**Graphical Abstract:**
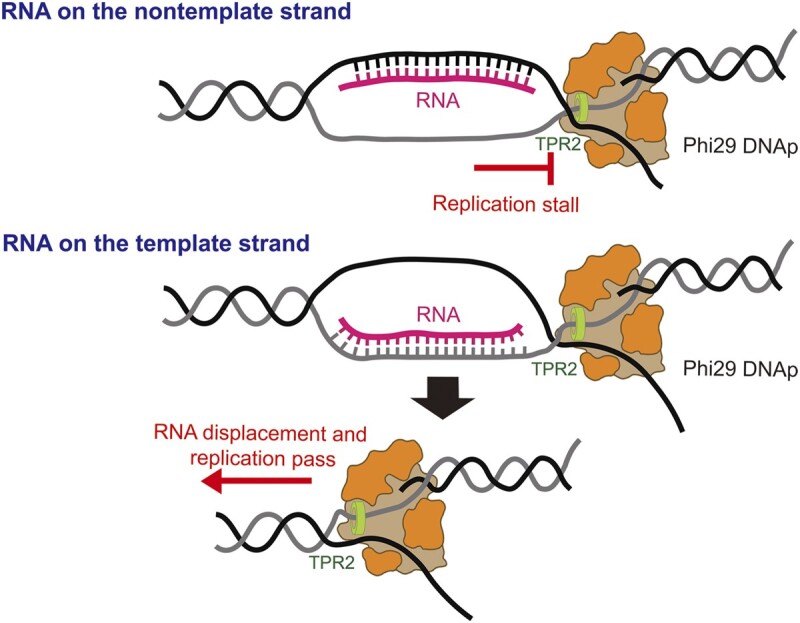

## Introduction

R-loops are triple-stranded nucleic acid structures consisting of an RNA–DNA hybrid and a displaced single-stranded DNA (ssDNA) (1,2). R-loops are generated when RNA transcripts anneal back into the template DNA strand (3). R-loops play important roles in diverse cellular activities including gene expression, chromosome segregation, immunoglobulin class switching, telomere regulation and immune activation (4–9). However, the misregulated accumulation of R-loops results in imperfect transcription and transcription–replication conflicts (TRCs), leading to genomic instability (10,11). R-loops hinder the progression of the replication fork, resulting in replication stress (10,12–14), and the displaced ssDNA becomes susceptible to endonucleases (15,16). Biochemical studies using a purified *Escherichia coli* (*E. coli*) DNA replication system demonstrated that R-loops on the leading strand cause transient replication stalling while they do not have a significant impact on the lagging strand. The *E. coli* replisome can synthesize DNA by bypassing R-loops (17). Using a yeast replisome system, it was reported that R-loops perturb fork progression in both head-on (HO) and co-directional (CD) collisions (18). A recent native electron microscopy (EM) study directly visualized TRCs mediated by R-loops, showing the bypass of R-loops by the replication fork (19). However, the molecular mechanism by which R-loops cause replication stalling remains elusive. Moreover, our understanding of the precise interactions between replication forks and R-loops remains limited. In this study, we conducted single-molecule imaging using the simple DNA replication system provided by the Phi29 DNA polymerase (Phi29 DNAp) to investigate the molecular mechanisms underlying replication fork stalling at R-loops.

**Figure 1.**
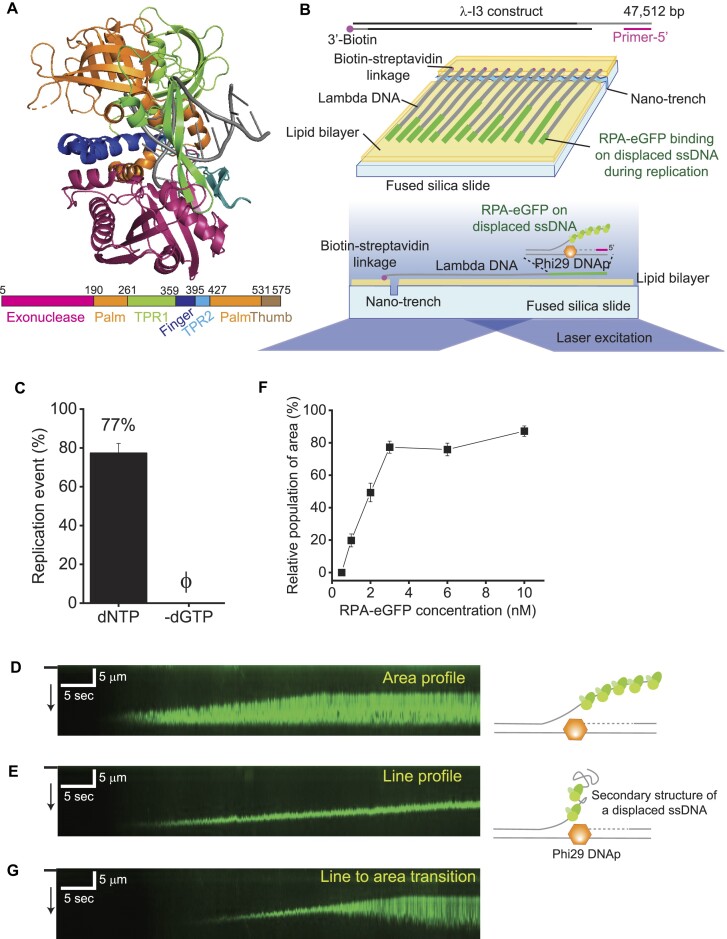
Replication of Phi29 DNAp. (**A**) Structure (PDB: 2PZS) and domains of Phi29 DNAp. (**B**) Schematic of a DNA curtain for Phi29 DNAp replication. Engineered lambda DNA (λ-I3) is modified with biotin and a primer for Phi29 DNAp replication at either end. In the DNA curtain, Phi29 DNAp synthesizes DNA from the primer unwinding duplex DNA. RPA–eGFP binds to the displaced non-template strand. RPA–eGFP is fluorescently imaged by TIRFM. (**C**) Phi29 DNAp replication efficiency. In the presence of all dNTPs, 77.3 ± 5.0% of DNA molecules display replication. In the absence of dGTP, no replication is shown. (**D**) A representative kymograph for (left) the area profile and (right) its schematic model. The black bar and arrow at the left indicate the barrier and flow direction, respectively. (**E**) A representative kymograph for (left) the line profile and (right) a schematic model. The black bar and arrow at the left indicate the barrier and flow direction, respectively. (**F**) Relative population of area species according to RPA concentration. Relative population is calculated by dividing the number of area species by the sum of area and line species. The error bars are obtained from the standard deviation (SD) in triplicate. (**G**) A kymograph for transition from line profile to area profile. The black bar and arrow at the left indicate the barrier and flow direction, respectively.

Phi29 DNAp is a single-subunit DNA polymerase of *Bacillus subtilis* bacteriophage Phi29 and belongs to B-family polymerases (Figure 1A) (20). Phi29 DNAp has high fidelity due to an exonuclease domain for proofreading (21,22). Structurally, the polymerase is comprised of a unique TPR2 domain, which encloses a primer–template junction and accommodates only ssDNA by forming a narrow channel together with thumb, palm and exonuclease domains (23,24). This configuration enables Phi29 DNAp to unwind duplex DNA without a separate helicase. In addition, the channel also bestows high processivity on Phi29 DNAp, bypassing the need for any auxiliary proteins such as a sliding clamp (23,24). Therefore, Phi29 DNAp is one of the simplest DNA replication systems. Because of its high fidelity and processivity, the polymerase has been extensively utilized for molecular biology approaches such as single-molecule sequencing and strand displacement amplification (25–28).

In this study, we directly visualized the DNA replication catalyzed by Phi29 DNAp using DNA curtain, a high-throughput single-molecule imaging technique that combines lipid fluidity, nano-fabrication, microfluidics and total internal reflection fluorescence microscopy (TIRFM) (29,30). We first characterized the biophysical properties of Phi29 DNAp at the single-molecule level. We then investigated the collision between replicating Phi29 and a single artificially inserted R-loop. We demonstrated that the single R-loop interrupts the Phi29 DNAp-dependent replication. When an RNA–DNA hybrid was formed on the non-template strand, replication was more severely stalled compared with the template strand. This result is consistent with previous TRC results showing that head-on TRCs are more deleterious than co-directional collisions. We revealed that secondary structures such as RNA–DNA hybrids and G-quadruplexes on the non-template strand impede the replication of Phi29 DNAp. Moreover, we observed the collision between Phi29 DNAp and RNA transcripts generated by T7 RNA polymerase (T7 RNAp). The RNA transcripts cause more stalling than the artificial R-loop because of the presence of RNAp. Our results provide insights into the principles of how replication stress is generated by R-loops that are also relevant for higher organisms.

## Materials and methods

### Sample preparation

Details of protein purification and DNA preparation are described in the Supplementary Information. The activity of purified Phi29 DNAp was compared with a commercial preparation (M0269, NEB) using rolling circle replication (RCR). The activity of purified replication protein A labeled with enhanced green fluorescent protein (RPA–eGFP) was tested by electrophoretic mobility shift assay with 30 nt ssDNA (31). Synthesized oligomers are listed in Supplementary Table S1.

### Circular dichroism

*In vitro* G-quadruplex (GQ) formation was measured using a spectropolarimeter (Jasco J-815). The GQ structures 1× human telomeric GQ (1× hTel_GQ), 2× hTel_GQ or the *cMyc* promoter at 10 μM were prepared in Phi29 DNAp buffer [50 mM Tris–HCl (pH 7.5), 10 mM MgCl_2_, 10 mM (NH_4_)_2_SO_4_ and 4 mM DTT (dl-dithiothreitol)] or Phi29 DNAp buffer supplemented with 50 mM KCl. Wavelengths from 220 to 340 nm were scanned at 50 nm/min. Ellipticity was averaged in triplicate.

To measure the melting temperature between RNA–DNA hybrid and DNA duplex, temperature-dependent circular dichroism was performed in Phi29 DNAp buffer. The temperature of the spectropolarimeter was changed from 25 to 98°C at a rate of 2°C/min, and the spectra between 220 and 300 nm were measured every 5°C.

### DNA curtain assay

Details for DNA curtain assays are described in the Supplementary Information. Briefly, flowcells with nano-trench patterns were prepared by following the previous protocol (32). A concentration of 2 nM of engineered lambda DNA (λ-I3) with biotin and a primer was pre-incubated with Phi29 DNAp at 30°C in Phi29 DNAp buffer [50 mM Tris–HCl (pH 7.5), 10 mM MgCl_2_, 10 mM (NH_4_)_2_SO_4_ and 4 mM DTT] supplemented with 0.1 mM dNTPs except for dGTP to attach Phi29 DNAp to the primer (33). Then the λ-I3 was anchored on the lipid bilayer via biotin–streptavidin linkage (Figure 1B). All DNA curtain experiments were conducted at room temperature (25°C). The DNA curtains were formed in imaging buffer [50 mM Tris–HCl (pH 8.8), 10 mM (NH_4_)_2_SO_4_, 10 mM MgCl_2_, 4 mM DTT, 3.2% glucose and 0.1× gloxy]. Replication in the curtain was started by switching from ‘imaging buffer’ to ‘replication buffer’ (imaging buffer supplemented with 3 nM RPA–eGFP and 0.1 mM dNTP) at a flow rate of 0.5 ml/min. For R-loop-containing GQ structures, 50 mM KCl was further supplemented to the replication buffer. The fluorescence signal was imaged by NIS-Element software (Nikon) for 6 min with 0.5 s/frame. For the replication resumption experiments at R-loops, 83 U/ml of RNase H (M0297L, NEB) was injected into the flowcell in replication buffer supplemented with 75 mM KCl to remove RNA.

### Collision between Phi29 DNAp and T7 transcripts

The detailed procedure is described in the Supplementary Information. Briefly, 0.3 nM lambda DNA (λ-DNA) containing T7 promoter was pre-incubated with 0.5 mM rNTPs, 2.5 μM Cy5-UTP (B8333, APExBIO) and 1 μl of T7 RNAp (M0251, NEB) in RNAp buffer [40 mM Tris–HCl (pH 8.8), 6 mM MgCl_2_, 1 mM DTT and 2 mM spermidine (124–20-9, Sigma)] for 5 min. Reactants were diluted in 200 μl of RNAp buffer and anchored on a lipid bilayer in the flowcell. To eliminate residual T7 RNAp, thermo-labile proteinase K (P8111, NEB) was added to the transcription reactants, and then the thermo-labile proteinase K was heat-inactivated at 55°C. Cy5-labeled transcripts were fluorescently imaged in RNAp buffer supplemented with 1.6% glucose and 0.1× gloxy under illumination with a 637 nm laser in DNA curtains with 0.1 s exposure time without shuttering.

### Image analyses

Imaging data were exported into TIFF format and analyzed by ImageJ software (NIH). Kymographs for each replication were made. Speed was analyzed by measuring the slope of the replication initiation point to end point. Processivity was analyzed by measuring the vertical distance between initiation and end points. Based on the kymographs, line and area species were counted, and then the relative population of area species was calculated by dividing the number of area species by the sum of area and line species.

## Results

### Real-time visualization of DNA replication conferred by Phi29 DNAp

We purified Phi29 DNAp and compared its DNA synthesis activity with that of a commercial preparation using RCR, and found equivalent synthesis activity (Supplementary Figure S1A, B). The replication behavior of Phi29 DNAp was examined using DNA curtains, comprised of engineered lambda DNA (λ-I3) (Figure 1B) (29,33). One end of λ-I3 was tagged with biotin for anchoring on the lipid bilayer, while the other end is open with the terminal single-stranded region bound to a primer for replication (Figure 1B). During DNA synthesis, Phi29 DNAp unwound duplex DNA and generated a displaced ssDNA, which was visualized by binding of RPA–eGFP under TIRFM (Figure 1B; Supplementary Figure S1C, D). DNA replication conferred by Phi29 DNAp in the presence of all four nucleotides was observed in real time by monitoring the extension of fluorescent lines, indicating RPA–eGFP binding to the displaced ssDNA (Supplementary Movie S1). About 77% of the λ-I3 molecules in the DNA curtains were replicated (Figure 1C). Given that replication as monitored by ‘line formation’ did not occur when dGTP was omitted, our assay reflects DNA replication conferred by Phi29 DNAp (Figure 1C; Supplementary Figure S1E). The starting position of the fluorescent lines was at ∼47 kbp, which is consistent with the primer position (∼47.5 kbp) in λ-I3 (Supplementary Figure S1F). We also tested a Phi29 DNAp deletion mutant (ΔTPR2), in which the TPR2 domain (amino acids 395–426) was deleted (Supplementary Figure S1A). The TPR2 domain is essential for helicase activity and high processivity of Phi29 DNAp (23,24). ΔTPR2 did not show any replication activity in our system as evidenced by the absence of any growing fluorescent lines (Supplementary Figure S1G). These data support that the fluorescence lines represent the replication of Phi29 DNAp. Unexpectedly, we observed two distinct types of replication (Figure 1D, E). One is a triangle-shaped kymograph (area profile), which results from the covering of RPA–eGFP of the whole displaced ssDNA as expected (Figure 1D). The other one is a line-shaped kymograph (line profile) (Figure 1E). We assumed that the line-shaped kymographs would result from incomplete RPA binding, which may be due to an inability to completely unfold secondary structures of the displaced ssDNA. This notion was tested by increasing the RPA–eGFP concentration. With increasing RPA–eGFP concentration, the area profile became more populated (Figure 1F). In addition, we observed that the line profile changed to an area profile during the replication because the unwinding of secondary structures by RPA–eGFP molecules appears to occur at different time points (Figure 1G).

### Biophysical properties of Phi29 DNAp

**Figure 2.**
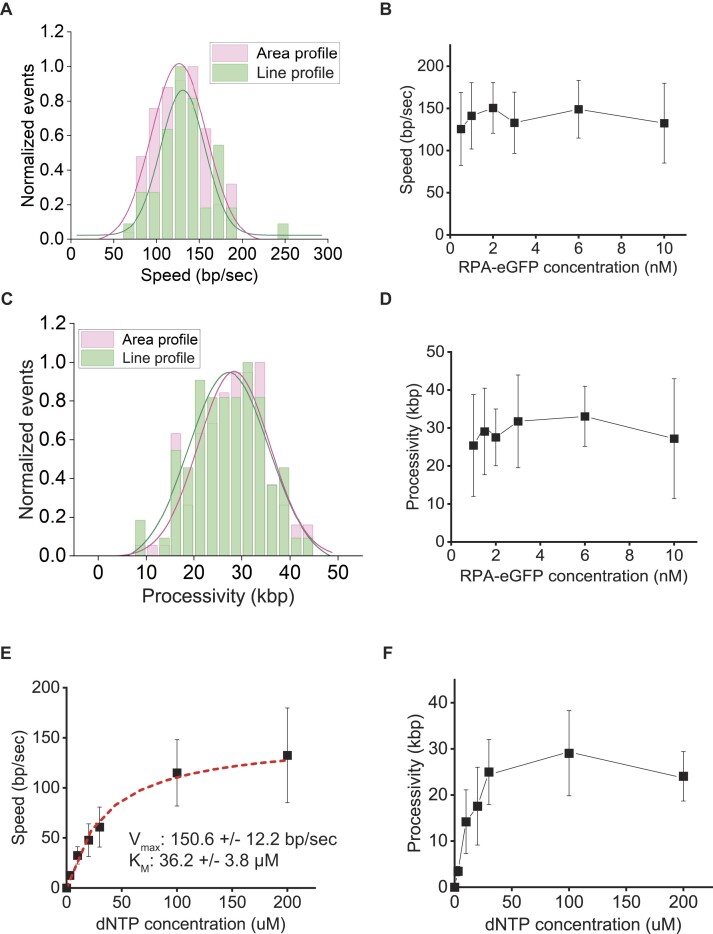
Speed and processivity of Phi29 DNAp. (**A**) Speed distributions for area (magenta) and line (green) profiles of Phi29 DNAp replication at 2 nM RPA. Each distribution is fitted with a single Gaussian function. The peak centers for area and line profiles are 127 ± 2 and 131 ± 3 bp/s, respectively, indicating little difference in the replication speed between area and line profiles. (**B**) Replication speed of Phi29 DNAp according to RPA–eGFP concentration. Errors are obtained from the width of a single Gaussian fit. (**C**) Processivity distributions for area (magenta) and line (green) profiles of Phi29 DNAp replication at 2 nM RPA. Each distribution is fitted with a single Gaussian function. The peak centers for area and line profiles are 28 ± 8 and 27 ± 10 kbp, respectively, indicating little difference in the processivity between area and line profiles. (**D**) Phi29 DNAp processivity according to RPA–eGFP concentration. The data and error bars are obtained from the single Gaussian fitting of (C). (**E**) Replication speed as a function of dNTP concentration. Data are fitted by the Michaelis–Menten equation. *V*_max_ (maximum replication speed) and *K*_M_ (Michaelis constant) were estimated as 150.6 ± 12.2 bp/s and 36.2 ± 3.8 μM, respectively. (**F**) Processivity as a function of dNTP concentration.

For both area and line profiles, Phi29 DNAp displayed linear movement with time until it stopped at a certain distance (Figure 1D, E). We did not observe transient pausing and backtracking, which were shown in the previous optical tweezers assays, because the spatiotemporal resolution of the DNA curtain is not sufficient (∼1 kbp/pixel and 0.5 s/frame) (34–36). From the kymographs, the replication speed and processivity of Phi29 DNAp were estimated at the single-molecule level (Figure 2A, B). At 2 nM RPA, where area and line profiles occurred in equal abundance, the replication speed at 0.1 mM dNTPs was ∼130 bp/s regardless of line and area profiles (Figure 2A). The speed did not change proportionally with RPA concentrations, indicating that RPA binding to the displaced ssDNA does not influence replication (Figure 2B). The replication speed we observed was slightly higher than in previous reports, probably because we used higher dNTP concentrations (34,35). Rapid replication also ensures that the hydrodynamic force (< 5 pN) by buffer flow does not disturb the replication, consistent with the previous result that DNA polymerization by Phi29 DNAp is not influenced by tension to DNA lower than 14 pN (35). The processivity was ∼30 kbp, which was independent of line and area species (Figure 2C, D). The processivity of Phi29 DNAp was smaller than the value (∼70 kbp) estimated from the previous bulk experiment (37). However, the bulk experiment did not completely exclude multiple turnovers of Phi29 DNAp and hence allowed for further elongation of DNA synthesis. Instead, taking into consideration the fact that the bacteriophage Phi29 genome is ∼19 kbp, the processivity we observed allows single Phi29 DNAp to replicate the entire Phi29 genome in most cases (38). We examined the speed and processivity in relation to dNTP concentrations. The dNTP-dependent speed followed Michaelis–Menten kinetics with a *V*_max_ of 150 bp/s and *a K*_M_ of 36 μM, compatible with previous measurements (Figure 2E) (36). On the other hand, the processivity reached ∼30 kbp at 30 μM dNTP and remained constant at higher dNTP concentrations (Figure 2F). This indicates that the processivity of Phi29 DNAp is not affected by the dNTP concentration. At dNTP concentrations <50 μM, Phi29 DNAp slowly replicated DNA and was unable to synthesize DNA up to 30 kbp within the given time window (Figure 2E). Hence, the processivity increased as the dNTP concentration increased.

We also tested bacterial (*E. coli*) ssDNA-binding protein (EcSSB) tagged with eGFP instead of RPA because Phi29 DNAp acts in bacteria (Supplementary Figure S2A). EcSSB displayed line and area profiles similar to those formed by RPA (Supplementary Figure S2B). The speed and the processivity in the presence of EcSSB were identical to those in the presence of RPA, indicating that the replication is not differentially affected by the ssDNA-binding proteins from different species (Supplementary Figure S2C, D). However, the intensity of the fluorescence signal of EcSSB was lower than that of RPA.

### Replication stalling at an R-loop with an RNA–DNA hybrid on the non-template strand

**Figure 3.**
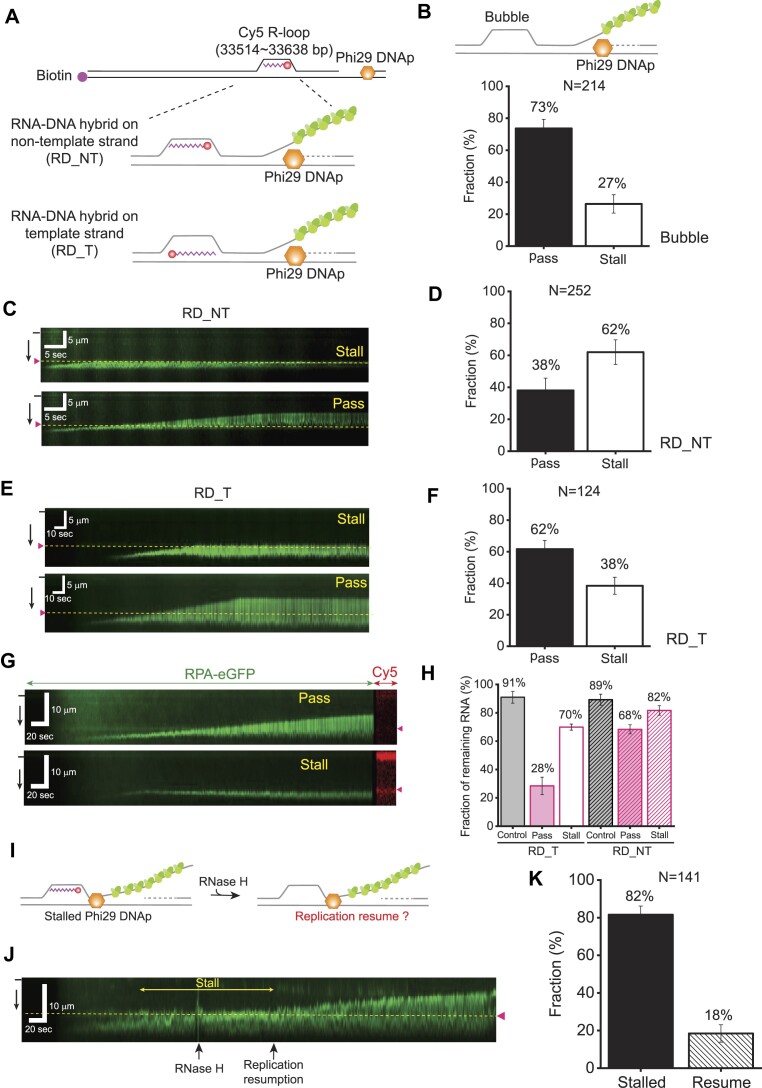
Collision between Phi29 DNAp and an R-loop. (**A**) Schematic of DNA curtain assay for the collision between Phi29 DNAp and an R-loop. Cy5-labeled RNA is annealed to either the non-template strand (RD_NT) or the template strand (RD_T) of Phi29 DNAp replication. Cy5 is labeled at the 3′ end of RNA to avoid unwanted replication from the RNA. (**B**) The collision of Phi29 DNAp and a bubble construct, which does not have an RNA–DNA hybrid. (Top) Schematic of the experiment and (bottom) pass and stall fractions. The total number of molecules analyzed (N) is 214. Error bars represent the SD in triplicate. (**C**) Kymographs for the collision events between Phi29 DNAp and an R-loop with an RNA–DNA hybrid on the non-template strand (RD_NT). Phi29 DNAp is either stalled at the R-loop (top) or passes the R-loop (bottom). The black bar and black arrow at the left represent the barrier and flow direction, respectively. The magenta arrowhead and yellow dashed line indicate the R-loop position. (**D**) Pass and stall fractions for the collision between Phi29 DNAp and an R-loop with RD_NT. The total number of molecules analyzed (N) is 252. Error bars represent the SD in triplicate. (**E**) Kymographs to show (top) stall and (bottom) pass of Phi29 DNAp for the collision with the R-loop of RD_T. The black bar and black arrow at the left represent the barrier and flow direction, respectively. The magenta arrowhead and yellow dashed line indicate the R-loop position. (**F**) Pass and stall fractions for the collision between Phi29 DNAp and an R-loop with RD_T. The total number of molecules analyzed (N) is 124. Error bars represent the SD in triplicate. (**G**) Kymographs for the fate of RNA after the collision between Phi29 DNAp and the R-loop with RD_NT. Cy5-labeled 39 nt RNA is fluorescently imaged after the replication reaction is complete. RNA disappears for passing Phi29 DNAp (top) whereas RNA remains for stalled Phi29 DNAp (bottom). The black bar and black arrow at the left represent the barrier and flow direction, respectively. The magenta arrowhead at the right indicates the R-loop position. (**H**) Fractions of the remaining 39 nt RNA at the R-loop after the collision with Phi29 DNAp. Control represents the fraction of remaining 39 nt RNA in the absence of Phi29 DNAp, which may reflect spontaneous dissociation or photobleaching of Cy5. The total number of molecules analyzed (N) for control, pass and stall of RD_T is 181, 80 and 31, respectively. The total number of molecules analyzed (N) for control, pass and stall of RD_NT is 91, 67 and 109, respectively. Error bars are obtained from the SD in triplicate. (**I**) Schematic for resumption of Phi29 DNA replication after RNA removal by RNase H treatment. (**J**) Representative kymograph showing the resumption of Phi29 DNAp stalled at an R-loop when the RNA strand at the R-loop is removed by RNase H. The black bar and black arrow at the left indicate the barrier and flow orientation, respectively. The yellow dashed line along with the magenta triangle shows the R-loop position. (**K**) Relative fractions for the replication resumption after RNA elimination. The total number of molecules analyzed (N) is 141. Error bars are obtained from the SD of binomial distribution.

We next examined the collision of replicating Phi29 DNAp with an R-loop (Figure 3A). A single R-loop was artificially inserted into a specific location of λ-I3, which has seven nickase sites between base pairs 33 514 and 33 638 (Supplementary Figure S3A) (29,33). R-loop formation was ensured by restriction enzyme digestion and the presence of a fluorescence signal of Cy5-labeled RNA in the DNA curtain (Supplementary Figure S3B, C). The Cy5 label was placed at the 3′ end of the RNA to prevent unintended synthesis from RNA by Phi29 DNAp. Cy5 fluorescence disappeared within 3 min after RNase H treatment, leading to RNA degradation, ensuring the R-loop formation (Supplementary Figure S3D). Prior to the collision with the R-loop, we tested a bubble structure without RNA–DNA hybrid (Figure 3B). As expected, 73% of Phi29 DNAp molecules passed the bubble (Figure 3B; Supplementary Figure S4A); 27% of stalling would result from any defects in bubble formation such as improper hybridization or ligation during λ-I3 preparation. Indeed, when the gap formed on λ-I3 was annealed with a complementary DNA oligomer, the stalling fraction was 24%, equivalent to that for the bubble, showing that, as expected, a mismatched sequence did not provide a major obstacle (Supplementary Figure S4B). In addition, when we removed the RNA strand from the R-loop, the stalling fraction was 27%, which was comparable with that for the bubble (Supplementary Figure S4C). RPA molecules bound to ssDNA of R-loops or bubbles (Supplementary Figure S4D–F). Even in the presence of RPA, the passing fraction was high for a bubble, suggesting that RPA binding to the ssDNA either in bubbles or of R-loops does not hinder the replication progression.

We then investigated the collision between replicating Phi29 DNAp and the artificial single R-loop. The RNA strand in the R-loop was placed on either the template or the non-template strand (Figure 3A). When Phi29 DNAp encountered the R-loop, Phi29 DNAp passed or stalled at the R-loop. We also observed a transient pause at the R-loop followed by replication progression (Supplementary Figure S4G). The transient pausing events were rare (∼7%, 6 out of 85) and the pause time was 36 ± 20 s. We scored these transient pauses as pass events because Phi29 DNAp actually passed the R-loop. Interestingly, the extent of stalling was dependent on which strand the RNA was annealed. When the RNA–DNA hybrid was formed on the non-template strand (RD_NT), ∼62 ± 5% of Phi29 DNAp exhibited stalling, whereas ∼38 ± 5% of Phi29 DNAp showed stalling for the RNA–DNA hybrid on the template strand (RD_T) (Figure 3C–F). The stalling fraction for RD_NT was higher than the background stalling (∼27 ± 3%), and the difference was statistically effective (*P*-value < 0.001). In contrast, the stalling fraction for RD_T was not statistically different from the background stalling (*P*-value ∼ 0.31). These results clearly showed that the presence of a single R-loop can impede the Phi29 DNAp replication. However, the positioning of the RNA–DNA hybrid on the non-template strand (RD_NT) represented a significant barrier to replication, while the presence of the hybrid on the template strand (RD_T) does not exert the same inhibitory effect. Furthermore, we tested the collision between Phi29 DNAp and an R-loop at a high RPA concentration (20 nM), where line species were suppressed. Even at this elevated RPA concentration, we observed no significant alternation in the stalling fraction. This result strongly suggested that RPA concentration does not affect Phi29 DNAp behavior at the R-loop (Supplementary Figure S4H, I).

### RNA remains on the R-loop when the replication stalls

**Figure 4.**
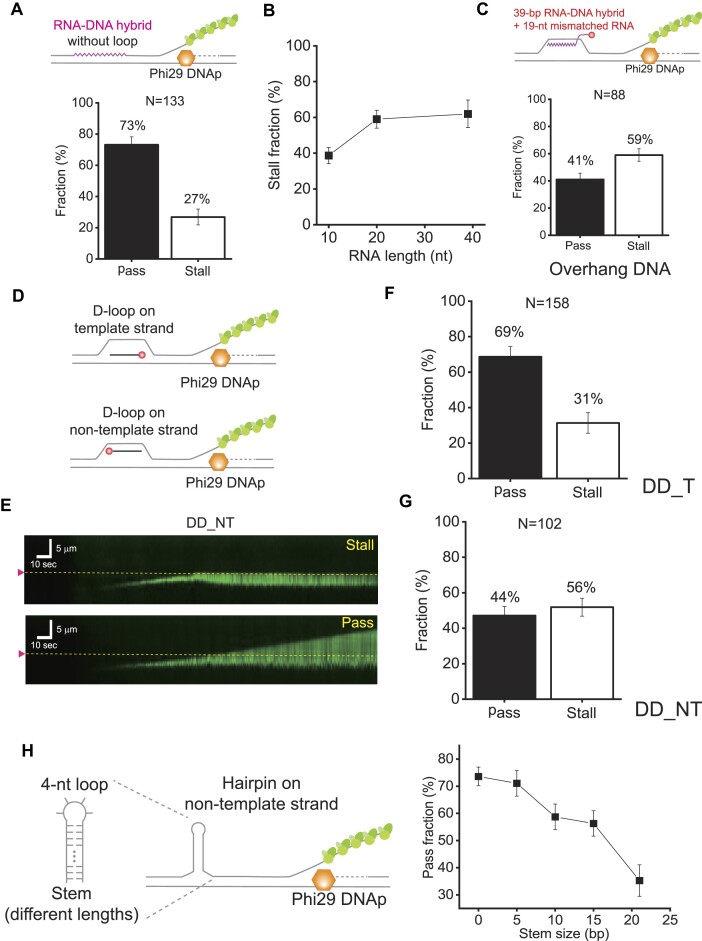
Replication stalling mechanism at an R-loop. (**A**) (Top) Schematic of an RNA–DNA hybrid inserted into the non-template strand. (Bottom) Pass and stall fractions of Phi29 DNAp in the presence of the RNA–DNA hybrid at the non-template strand. The total number of molecules analyzed (N) is 133. Error bars represent the SD in triplicate. (**B**) Stall fraction of Phi29 DNAp according to the size of RNA at the RD_NT R-loop. (**C**) Collision of Phi29 DNAp with the RD_NT R-loop having a 3′ overhang of RNA, which formed a 39 bp RNA–DNA hybrid with 19 nt mismatches at the 3′ end. The total number of molecules analyzed (N) is 88. Error bars represent the SD in triplicate. (**D**) Schematic of collision of Phi29 DNAp with a D-loop, which had 39 nt DNA on either the template strand (DD_T) or the non-template strand (DD_NT). Cy5 is labeled at the 3′ end to avoid unwanted replication from the D-loop DNA. (**E**) Kymographs for the collision events between Phi29 DNAp and a D-loop with a DNA–DNA hybrid on the non-template strand (DD_NT). Phi29 DNAp is either stalled at the D-loop (top) or passes the D-loop (bottom). The magenta arrowhead and yellow dashed line indicate the D-loop position. (**F**) Pass and stall fractions for a D-loop with DD_T. The total number of molecules analyzed (N) is 158. Error bars represent the SD in triplicate. (**G**) Pass and stall fractions for the D-loop with DD_NT. The total number of molecules analyzed (N) is 102. Error bars represent the SD in triplicate. (**H**) (Left) Schematic of collision of Phi29 DNAp with a hairpin on the non-template strand. The hairpin consists of a 4 nt loop and a stem with different lengths (5, 10, 15 and 21 bp). (Right) Pass fraction as a function of stem sizes of hairpin structures. The data point at 0 stem is adopted from the pass fraction of a bubble structure in Figure 3B. The number of molecules analyzed for each data point is >100. The error bar represents the SD in triplicate.

We next examined whether the R-loop remained intact following the collision with Phi29 DNAp or if the RNA strand was displaced. As RNA was labeled with Cy5, a 637 nm laser was illuminated to monitor the RNA after the collision (Figure 3G, H). In the absence of Phi29 DNAp, ∼10% of RNA disappeared, leaving 90% remaining, presumably due to Cy5 photobleaching and/or spontaneous RNA dissociation. In the case of RD_NT with 39 nt RNA, when Phi29 DNAp was stalled, 82% of the RNA remained at the R-loop. When Phi29 DNAp passed through the R-loop, 68% of the RNA still remained. These findings indicated that Phi29 DNAp does not disrupt the RNA in the RD_NT. For RD_T, when Phi29 DNAp was stalled at the R-loop, a substantial portion of the RNA (70%) survived. Taken together with the RD_NT case, our results demonstrated that the stalled Phi29 DNAp does not lead to RNA displacement at R-loops. In stark contrast, when Phi29 DNAp passed the RD_T R-loop, the remaining RNA fraction was dramatically reduced (28%), indicating that Phi29 DNAp displaces RNA or unwinds an RNA–DNA hybrid on the template strand when it passes the R-loop. This is consistent with our result from an RNA–DNA hybrid experiment in Figure 4A. On the other hand, there was still 28% RNA left even in the case of Phi29 DNAp passage for RD_T, implying that replication bypasses through R-loops as shown in previous studies (17,19,39). For the R-loop with 20 nt RNA, we had similar results (Supplementary Figure S5A, B). We also examined if the stalled replication resumes when the RNA is removed by RNase H (Figure 3I). RNase H eliminated almost all RNA molecules from R-loops within 3 min (Supplementary Figure S3D). Upon injecting RNase H after replication initiation, we observed the resumption of stalled Phi29 DNAp, indicating that the R-loop structure indeed acts as a blockage to the progression of replication (Figure 3J, K). The relatively small fraction of resumption could be attributed to the dissociation of the stalled Phi29 DNAp.

### The R-loop structurally hinders the replication progression

We explored how a single R-loop blocks the replication of Phi29 DNAp. First, we tested if Phi29 DNAp can synthesize DNA when passing through an RNA–DNA hybrid (Figure 4A). The fraction of passing polymerases through an RNA–DNA hybrid (73%) was as high as that for a bubble, indicating that RNA–DNA hybrids do not impede the replication of Phi29 DNAp. This accounts for the high passing fraction for RD_T. We next reduced the size of RNA. For 10 nt RNA, the stalling fraction was reduced to 40% whereas 20 nt RNA showed a similar stalling fraction to 39 nt RNA (Figure 4B; Supplementary Figure S6A, B), suggesting that RNA size affects the replication stalling but has little effect beyond a certain size for the artificial single R-loop. We then tested an R-loop in which RNA at the 3′ end was partially mismatched to DNA (Figure 4C; Supplementary Figure S6C). The mismatched 3′ overhang did not change the stalling fraction (59% for the 39 nt RNA–DNA hybrid with a 19 nt mismatch and 64% for the 20 nt RNA–DNA hybrid with a 19 nt mismatch). To check if the stalling for RD_NT is specific to the RNA–DNA hybrid, we examined a D-loop, in which DNA was annealed to either the template (DD_T) or non-template strand (DD_NT) (Figure 4D). Similar to the R-loop, DD_NT showed a higher stalling fraction (∼56%) compared with DD_T (∼31%) (Figure 4E–G; Supplementary Figure S6D), indicating that RNA or DNA placed on the non-template strand has a tendency to impede replication. On the other hand, the overall stalling fraction for the D-loop was 6–7% lower than that for the R-loop. This difference could be potentially attributed to the difference of thermodynamic stability between a RNA–DNA hybrid and double-stranded DNA. The thermodynamic stability of RNA–DNA and DNA–DNA depends on the sequences (40,41). To check the thermodynamic stability of the RNA–DNA hybrid and a DNA duplex, we measured the temperature-dependent cicular dichroism for each substrate (Supplementary Figure S7A, B). As the temperature increased, the cicular dichroism spectra of both 39 bp RNA–DNA hybrid and DNA duplex gradually changed and abruptly changed at high temperature. When the change of peak at 265 nm was monitored, the melting temperatures of the RNA–DNA hybrid and the DNA duplex were 82.7 and 83.8°C, respectively, indicating that there is no thermodynamic difference between 39 bp DNA duplex and 39 bp RNA–DNA hybrid (Supplementary Figure S7C). These results suggest that the difference of stalling fractions for the R-loop and D-loop is not attributed to the thermodynamic stability of the RNA–DNA hybrid or DNA duplex in the loop structures. In the future, further study is required to understand why the D-loop showed a lower stalling fraction than the R-loop.

Based on the above results, we assumed that a secondary structure formed on the non-template strand leads to the stalling of Phi29 DNAp during replication. To prove this, we tested hairpin structures with a 4 nt loop and a stem with different stem lengths (Figure 4H). The pass fraction of a hairpin with a 5 bp stem is similar to that of a bubble (0 bp stem), showing that a 5 bp stem hairpin does not impede the Phi29 DNAp replication. However, with an increase of the stem length, the pass fraction decreased. These results support our assumption that the presence of secondary structures on the displaced non-template strand hinders the Phi29 DNAp replication. In addition, it is worth noting that there is a noticeable reduction of the pass fraction between 5 bp and 10 bp stems, suggesting that a hairpin with a 10 bp stem, at minimum, can block the Phi29 replication.

### G-quadruplex formation at the displaced ssDNA enhances replication stalling

**Figure 5.**
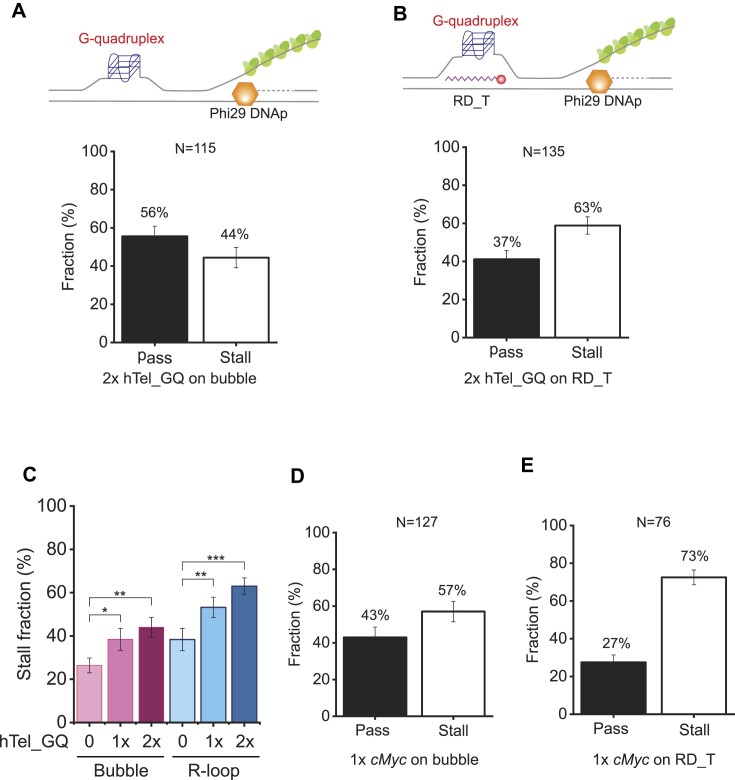
G-quadruplex (GQ) effect on replication stalling at an R-loop. (**A**) Pass and stall fractions for 2× hTel_GQ in a bubble without RNA–DNA hybrid at 50 mM K^+^. The schematic is shown above. The total number of molecules analyzed (N) is 115. Error bars represent the SD in triplicate. (**B**) Pass and stall fractions for 2× hTel_GQ for the RD_T R-loop at 50 mM K^+^. The schematic is shown above. The total number of molecules analyzed (N) is 135. Error bars represent the SD in triplicate. (**C**) Stall fractions for hTel_GQs in a bubble and an R-loop (RD_T) at 50 mM K^+^. *P-value ≤ 0.05, **P-value ≤ 0.01 and ***P-value ≤ 0.001. (**D**) Pass and stall fractions for 1× *cMyc* GQ in a bubble without RNA–DNA hybrid at 50 mM K^+^. The total number of molecules analyzed (N) is 127. Error bars represent the SD in triplicate. (**E**) Pass and stall fractions for 1× *cMyc* GQ for the RD_T R-loop at 50 mM K^+^. The total number of molecules analyzed (N) is 76. Error bars represent the SD in triplicate.

When the displaced ssDNA is guanine rich, it can form GQ structures, which facilitate R-loop formation and stabilize R-loops (42–46). We examined the effect of GQ formation at the displaced ssDNA for RD_T on the Phi29 DNAp replication because RD_T displayed a reduced stalling fraction compared with RD_NT. To this end, when a bubble was formed during the λ-DNA preparation, oligomers that contained 1× hTel_GQ, 2× hTel_GQ or the *cMyc* promoter sequence were used (Supplementary Table S1). Hence, the displaced ssDNA of RD_T contained GQ structures such as 1× hTel_GQ, 2× hTel_GQ or the *cMyc* promoter (Figure 5A, B). The formation of GQs in Phi29 DNAp buffer was verified by cicular dichroism measurements (Supplementary Figure S8A, B). Interestingly, even in the absence of K^+^, human telomeric sequences and the *cMyc* promoter formed GQ structures in the replication buffer because the replication buffer contained 10 mM ammonium ions, which can stabilize GQ formation even at low concentration (Supplementary Figure S8A, B) (47). For 1× hTel_GQ, the dichroism spectra did not display typical hybrid conformation in Phi29 DNAp buffer even in the presence of 50 mM K^+^ because Phi29 DNAp buffer contained 10 mM MgCl_2_ and 10 mM (NH_4_)_2_SO_4_, which can induce other GQ conformations (48–50). The 2× hTel_GQ showed the hybrid conformation, which can form double stacks of GQs in the presence of 50 mM K^+^ (Supplementary Figure S8A) (51,52). On the other hand, the *cMyc* promoter sequence, which is also known to form a GQ structure, displayed a typical dichroism spectrum in Phi29 DNAp buffer regardless of K^+^ (Supplementary Figure S8B) (53–56). RPA–eGFP did not bind 1× hTel_GQ even at 30 nM, as reported previously, and hence GQs in our experiments were not disrupted by 2 nM RPA–eGFP (Supplementary Figure S8C) (57). We first examined the effect of GQs on replication without the RNA–DNA hybrid. When GQ was formed on the displaced ssDNA, the stalling fraction was increased, showing that GQ by itself has an inhibitory effect on the replication (Figure 5A, C; Supplementary Figure S8D). This result is consistent with previous studies showing that GQs block the replication *in vitro* and *in vivo* (58,59). When GQs were formed on the displaced ssDNA of RD_T, 1× hTel_GQ and 2× hTel_GQ increased the stalling fraction by 1.4 and 1.8 times, respectively (Figure 5B, C; Supplementary Figure S8E). In particular, 2× hTel_GQ increased the stalling fraction up to 63%, which was comparable with that of RD_NT. This increase of stalling for RD_T with 2× hTel_GQ was due to the synergistic effect of R-loop and GQ formation. Even in the absence of K^+^, the human telomeric sequence still blocked the progression of Phi29 DNAp at the bubble because it could form GQ structure in the replication buffer (Supplementary Figure S8A, F). We next tested the promoter of the *cMyc* gene, which was also inserted into λ-I3. *cMyc* GQ alone blocked the replication of Phi29 DNAp in up to 57% of all cases (Figure 5D). When *cMyc* GQ was formed on the displaced ssDNA of RD_T, the stalling fraction increased up to 73%, which was higher than 2× hTel_GQ (Figure 5E). We believe that this dramatic increase results from higher thermodynamic stability of *cMyc* GQ compared with hTel_GQs. *cMyc* GQ has a melting temperature (*T*_m_) of 84°C and a free energy difference (Δ*G*) between unfolded and folded conformations at 100 mM K^+^ of 7.6 kcal/mol, whereas for 1× hTel_GQ these values are 66°C and 4.7 kcal/mol (55,60,61). Taken together, our results demonstrated that GQ formation at the displaced ssDNA of an R-loop enhances the stalling of the progression of replication. Moreover, the thermodynamic stability of GQ influences the stalling. These results strongly support our claim that secondary structures of the non-template strand block the replication of Phi29 DNAp.

### Collision of Phi29 DNAp with transcription of T7 RNAp

**Figure 6.**
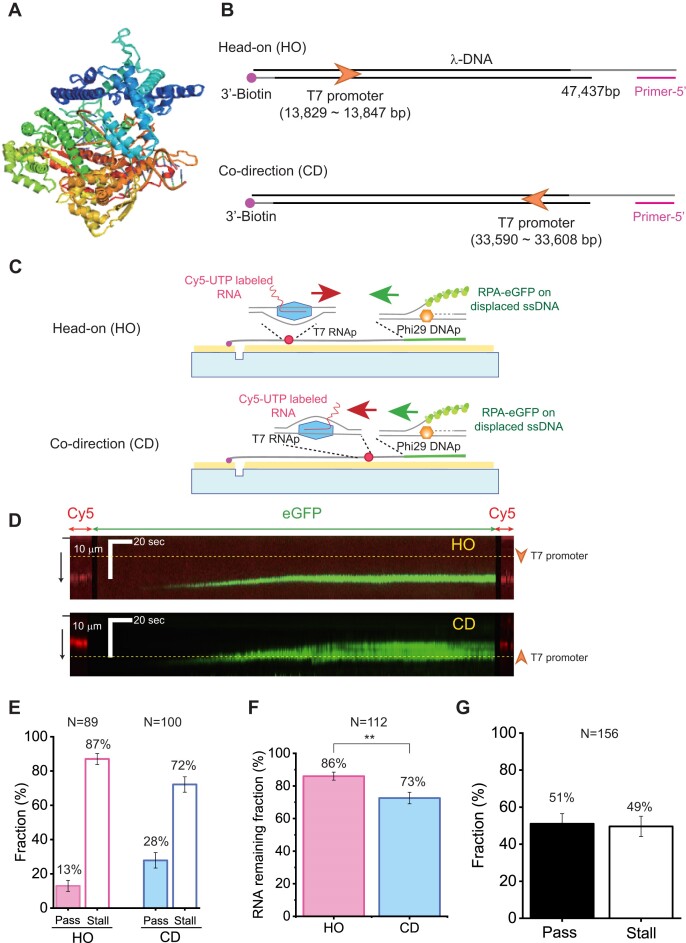
Collision of Phi29 DNAp with RNA transcripts. (**A**) Structure of T7 RNAp (PDB: 1H38). (**B**) Schematic of λ-DNA for the collision between Phi29 DNAp and RNA transcripts of T7 RNAp. The T7 promoter is inserted between base pairs 13 829 and 13 847 of λ-DNA. Depending on which end of the λ-DNA has biotin and the primer, (top) HO and (bottom) CD collisions are determined. (**C**) Schematic of the DNA curtain assay for (top) HO collision and (bottom) CD collision of Phi29 DNAp and RNA transcripts generated by T7 RNAp. (**D**) Kymographs for the collision between Phi29 DNAp and RNA transcripts of T7 RNAp: (top) HO collision and (bottom) CD collision. Cy5-labeled RNA transcript and RPA-eGFP for replication are denoted as red and green arrows above, respectively. The black bar and black arrow on the left represent the barrier and flow direction, respectively. The yellow dashed line and the orange arrowhead on the right indicate the position and orientation of the T7 promoter that is inserted into the λ-DNA, respectively. (**E**) Pass and stall fractions for the collisions of Phi29 DNAp and RNA transcripts without removal of T7 RNAp. N represents the number of analyzed molecules. The error bars are SDs in triplicate. (**F**) RNA remaining fractions for the stalled Phi29 DNAp. N represents the number of analyzed molecules. Error bars are obtained from the SD of binomial distribution. **P-value ≤ 0.01. (**G**) Pass and stall fractions for the collision of Phi29 DNAp and T7 RNAp bound at a T7 promoter site in the absence of rNTPs. N represents the number of analyzed molecules. The error bars are the SD in triplicate.

We also investigated how transcribing RNAp affects replication using T7 RNAp. T7 RNAp was used to generate fluorescent RNA transcripts with Cy5-labeled UTP (Figure 6A; Supplementary Figure S9A). To produce RNA transcripts by T7 RNAp on λ-DNA, we inserted the T7 promoter sequence into λ-DNA (Figure 6B). RNA transcripts produced by T7 RNAp in the λ-DNA were fluorescently visualized in DNA curtains (Supplementary Figure S9B). We then examined the collision between Phi29 DNAp and T7 RNAp transcripts. Depending on the positions of biotin and the primer, HO and CD collisions between Phi29 DNAp and T7 RNAp were chosen (Figure 6B, C). In the case that T7 RNAp was not removed, Phi29 DNAp mostly stalled at RNA transcripts (Figure 6D, E). The stalling fractions were 87% and 72% for HO and CD collisions, respectively. HO collision induced more stalling events than CD collision (Figure 6E). We then examined whether or not RNA transcripts remained at the site of the collision. When Phi29 DNAp was stalled at the transcripts, RNA transcripts mostly remained (86% for HO and 73% for CD), indicating that stalled Phi29 DNAp does not remove RNA transcripts (Figure 6F). In addition, the stalling fractions at the transcripts were higher than those at the artificial R-loop. Such an increase of stalling fractions could result from T7 RNAp remaining at the transcripts. To examine whether or not the remaining T7 RNAp affects the stalling, we heat-inactivated T7 RNAp or deproteinized the reaction using thermo-labile proteinase K, which is inactive when shifted to 55°C. In either case, we did not observe any Cy5-labeled RNA transcripts in DNA curtains after removing T7 RNAp (Supplementary Figure S9C, D). We thus speculated that RNA transcripts were also dissociated when T7 RNAp was removed. Instead, we examined the collision with T7 RNAp bound at the T7 promoter in the absence of rNTPs. Excessive T7 RNAp was incubated with λ-DNA containing a T7 promoter to ensure that the T7 promoter site was mostly occupied by T7 RNAp (Supplementary Figure S9E). T7 RNAp alone could impede the replication (49% stall) (Figure 6G). However, the effect of T7 RNAp alone is less than that of transcribing RNAp containing RNA transcripts, suggesting that the high stalling rate for transcribing RNAp is attributed to the presence of RNAp in RNA transcripts.

## Discussion

### Speed and processivity difference from previous results

The replication speed of Phi29 DNAp from our single-molecule experiments is ∼130 bp/s, which is higher than that of previous bulk experiments (<33 bp/s) (62,63). This discrepancy primarily results from the low dNTP concentration of the previous bulk experiments, in which the dNTP concentration was 20 μM. At 20 μM, our replication rate is ∼50 bp/s, which is comparable with the previous bulk results (Figure 2E). In addition, in the previous assays, the binding and DNA polymerization of Phi29 DNAp and Phi29 terminal protein (TP) were not separated. In contrast, our single-molecule experiments allowed us to differentiate between polymerase binding and DNA synthesis, providing a more accurate measurement of the replication speed. The replication speed of Phi29 DNAp was also assessed using optical tweezers at the single-molecule level (34,35). Under low tension to DNA (<10 pN) at 50 μM dNTP, the replication speed was ∼80 bp/s, which is comparable with that in our study (Figure 2E). On the other hand, our observed processivity (∼30 kbp) is 2.3 times lower than the estimation obtained from the previous bulk replication assays (∼ 70 kbp) (Figure 2F) (37). The earlier experiments adopted RCR with primed M13 closed-circular ssDNA, which did not represent a single-turnover reaction. In contrast, our single-molecule measurements captured the DNA synthesis of the polymerase without free polymerases, ensuring a single-turnover replication. Hence, our determination of processivity is more reliable. Despite the observed decrease in the processivity, our measured processivity is still sufficient to encompass the entire genome of Phi29 DNA (∼19 285 bp), indicating that a single round of replication can replicate the Phi29 genome in the vast majority of cases (38).

### Stalling efficiency at the collision with an R-loop depends on the position of RNA–DNA hybrid

We tested the collision between Phi29 DNAp and a single R-loop without RNAp. We found that the R-loop with RD_NT inhibited the Phi29 replication more severely compared with RD_T. To investigate why RD_NT blocks the replication more strongly than RD_T, we checked the effect of RNA–DNA hybrids, which are structurally and thermodynamically different from DNA homoduplexes. Phi29 DNAp demonstrated the ability to synthesize DNA traversing an RNA–DNA hybrid, indicating that Phi29 DNAp can displace the RNA–DNA hybrids during replication (Figure 4A). To reduce the thermodynamic stability of the RNA–DNA hybrid, we shortened the length of the RNA within the R-loop (Figure 4B). When the RNA–DNA hybrid was reduced to 20 bp, the stalling fraction did not change. Instead, with a further reduction to a 10 bp RNA–DNA hybrid, the stalling fraction decreased to ∼40%, which was not as dramatic as anticipated. Similarly, the 3′-mismatched RNA also blocked the replication at the same rate as RD_NT. These findings suggest that the thermodynamic stability of the RNA–DNA hybrid does not play a critical role in the replication stalling. Subsequently, we tested a D-loop configuration. Similar to the R-loop configuration, DD_NT exhibited a stronger hindrance on Phi29 DNAp than DD_T. The stalling fraction was slightly lower for the D-loop than for the R-loop even though the thermodynamic stability of DNA duplex was similar to that of the RNA–DNA hybrid (Figure 4F, G; Supplementary Figure S7C). These results demonstrate that the replication stalling observed for RD_NT is not solely attributed to the presence of an RNA–DNA hybrid.

We then hypothesized that a secondary structure in the displaced ssDNA or the non-template strand hinders the replication of Phi29 DNAp. To verify the hypothesis, another secondary structure, a hairpin configuration on the non-template strand, was tested. The hairpin also inhibited the progression of replication, demonstrating that formation of a secondary structure on the non-template strand such as an RNA–DNA hybrid and DNA duplex stalls Phi29 DNAp-mediated replication. Our claim is also supported by our GQ experiments. The addition of GQ structures to the non-template strand increased the stalling efficiency of RD_T by 1.7 times, implying that the nucleic acid structures on the non-template strand would interfere with Phi29 DNAp. Why does a secondary structure interfere with the Phi29 DNAp-mediated replication? Structurally, the strand displacement activity comes from the sharp bend (∼90°) of the template strand inside the Phi29 DNAp and the steric exclusion of the non-template strand at the narrow channel that is formed by the TPR2, palm and finger domains, together with the exonuclease subdomains (20). A single-molecule optical tweezers assay reported that when the sharp template bending was inhibited under tension, the strand displacement was impeded, and Phi29 DNAp underwent a long pause (35). When a secondary structure such as an RNA–DNA hybrid, DNA–DNA annealing, GQ or a hairpin is formed at the non-template ssDNA, it reduces the flexibility of displaced ssDNA at the junction and prevents the template strand from bending sharply, and hence Phi29 DNAp may stall. Many helicases including replicative ones require the sharp bending of the template strand to unwind duplex DNA (64,65). Besides the direct impact of a secondary structure on the non-template strand, there is a possibility that the secondary structure indirectly influences the template strand. Such an indirect effect could play a role in shaping the behavior of Phi29 DNAp. On the other hand, we cannot exclude the possibility that Phi29 DNAp can have two conformations: one for a stationary mode and another for a progressive mode needed for DNA synthesis. When the Phi29 DNAp encounters a R-loop or D-loop, it might change the conformation from a progressive mode to a stationary mode. The speculations can be verified by other biophysical studies such as single-molecule FRET.

### Collision of Phi29 DNAp with RNA transcripts of T7 RNAp

We examined the collision between Phi29 DNAp and RNA transcripts generated through ongoing T7 RNAp, which was more biologically relevant. The collision with the RNA transcripts led to higher stalling fractions compared with the artificial R-loop (Figure 6E). Such an increase can be attributed to the remaining T7 RNAp. We attempted to remove solely T7 RNAp. However, when T7 RNAp was removed, RNA transcripts were equally dissociated. Structurally, the 8 bp RNA–DNA hybrid formed as part of the transcription bubble is thermodynamically unstable (Figure 6A). Hence, the removal of T7 RNAp may induce the dissociation of RNA transcripts. Instead, we tested T7 RNAp bound to the T7 promoter, which also blocked the replication. Our results suggest that the remaining T7 RNAp at an RNA transcript plays an important role in the replication stalling. This is consistent with the finding that RNA mostly remained when Phi29 DNAp was stalled because T7 RNAp was not displaced by Phi29 DNAp (Figure 6F).

In addition, HO collision exhibited a higher stalling fraction (∼80%) than CD collision (∼70%). The HO and CD configurations were identical to RD_NT and RD_T, respectively. Therefore, the difference between HO and CD appears to come from the location of the RNA–DNA hybrid. Consistent with our results, many previous studies about TRCs reported that HO collision is more deleterious than CD collision (66,67). On the other hand, in a previous study using an *E. coli* replication system, CD collision caused more stalling than HO collision, suggesting that R-loops in the lagging strand cause little stalling (17). Because *E. coli* replicative helicase moves along the lagging strand, the CD collision is similar to the configuration of RD_T in our experiments, giving less stalling.

### G-quadruplex formation at a R-loop enhances the replication stalling

We demonstrated that GQ structures formed at the displaced ssDNA of a single R-loop enhance replication stalling. Moreover, multiple GQs more strongly block the replication. It was reported that GQs and R-loops are formed in human telomeres consisting of tandem repeats of d(TTAGGG) (68–70). Human telomeric GQ can be stacked by forming hybrid structures in the presence of K^+^. On the other hand, telomeric R-loops derived from non-coding RNA TERRA (telomeric repeat-containing RNA) are involved in telomere maintenance and alternative lengthening of telomeres (ALT) (69,71). Our results imply that GQs at R-loops synergistically cause replication stress at telomeres, which is known as a hallmark of ALT cells (70). On the other hand, we tested the promoter sequence of the *cMyc* gene, which is also known to form a GQ structure and is involved in gene regulation (55,56). For *cMyc* GQ, the stalling fraction is higher than for hTel_GQs. The *cMyc* GQ (Δ*G*: 6–8 kcal/mol) is more stable than 1× hTel_GQs (∼3 kcal/mol) (55,60,72). The increased stalling conferred by *cMyc* GQ results from the high thermodynamic stability of the GQ structure. This is consistent with our data which revealed that 2× hTel_GQ showed more stalling than 1× hTel_GQ. Our results show that the thermodynamic stability of GQs influences the stalling of replication at R-loops. Furthermore, our results imply that the stable secondary structures at the displaced ssDNA enhance the R-loop stability (43).

## Supplementary Material

gkad1101_Supplemental_Files

## Data Availability

The data underlying this article are available in the article and in its online supplementary material.

## References

[1] Hegazy Y.A. , Fernando C.M., Tran E.J. The balancing act of R-loop biology: the good, the bad, and the ugly. J. Biol. Chem. 2020; 295:905–913. 31843970 10.1074/jbc.REV119.011353 PMC6983857

[2] Thomas M. , White R.L., Davis R.W. Hybridization of RNA to double-stranded DNA: formation of R-loops. Proc. Natl Acad. Sci. USA. 1976; 73:2294–2298. 781674 10.1073/pnas.73.7.2294 PMC430535

[3] Liu L.F. , Wang J.C. Supercoiling of the DNA template during transcription. Proc. Natl Acad. Sci. USA. 1987; 84:7024–7027. 2823250 10.1073/pnas.84.20.7024 PMC299221

[4] García-Muse T. , Aguilera A. R loops: from physiological to pathological roles. Cell. 2019; 179:604–618. 31607512 10.1016/j.cell.2019.08.055

[5] Ginno P.A. , Lott P.L., Christensen H.C., Korf I., Chédin F. R-loop formation is a distinctive characteristic of unmethylated human CpG island promoters. Mol. Cell. 2012; 45:814–825. 22387027 10.1016/j.molcel.2012.01.017 PMC3319272

[6] Kabeche L. , Nguyen H.D., Buisson R., Zou L. A mitosis-specific and R loop-driven ATR pathway promotes faithful chromosome segregation. Science. 2018; 359:108–114. 29170278 10.1126/science.aan6490 PMC5875943

[7] Roy D. , Yu K., Lieber M.R. Mechanism of R-loop formation at immunoglobulin class switch sequences. Mol. Cell. Biol. 2008; 28:50–60. 17954560 10.1128/MCB.01251-07 PMC2223306

[8] Pfeiffer V. , Crittin J., Grolimund L., Lingner J. The THO complex component Thp2 counteracts telomeric R-loops and telomere shortening. EMBO J. 2013; 32:2861–2871. 24084588 10.1038/emboj.2013.217 PMC3817467

[9] Crossley M.P. , Song C., Bocek M.J., Choi J.H., Kousorous J., Sathirachinda A., Lin C., Brickner J.R., Bai G., Lans H. et al. R-loop-derived cytoplasmic RNA–DNA hybrids activate an immune response. Nature. 2023; 613:187–194. 36544021 10.1038/s41586-022-05545-9 PMC9949885

[10] Aguilera A. , García-Muse T. R loops: from transcription byproducts to threats to genome stability. Mol. Cell. 2012; 46:115–124. 22541554 10.1016/j.molcel.2012.04.009

[11] Crossley M.P. , Bocek M., Cimprich K.A. R-loops as cellular regulators and genomic threats. Mol. Cell. 2019; 73:398–411. 30735654 10.1016/j.molcel.2019.01.024 PMC6402819

[12] Castellano-Pozo M. , García-Muse T., Aguilera A. R-loops cause replication impairment and genome instability during meiosis. EMBO Rep. 2012; 13:923–929. 22878416 10.1038/embor.2012.119 PMC3463965

[13] Gan W. , Guan Z., Liu J., Gui T., Shen K., Manley J.L., Li X. R-loop-mediated genomic instability is caused by impairment of replication fork progression. Genes Dev. 2011; 25:2041–2056. 21979917 10.1101/gad.17010011 PMC3197203

[14] Gómez-González B. , García-Rubio M., Bermejo R., Gaillard H., Shirahige K., Marín A., Foiani M., Aguilera A. Genome-wide function of THO/TREX in active genes prevents R-loop-dependent replication obstacles. EMBO J. 2011; 30:3106–3119. 21701562 10.1038/emboj.2011.206 PMC3160181

[15] Huertas P. , Aguilera A. Cotranscriptionally formed DNA:RNA hybrids mediate transcription elongation impairment and transcription-associated recombination. Mol. Cell. 2003; 12:711–721. 14527416 10.1016/j.molcel.2003.08.010

[16] Basu U. , Meng F.L., Keim C., Grinstein V., Pefanis E., Eccleston J., Zhang T., Myers D., Wasserman C.R., Wesemann D.R. et al. The RNA exosome targets the AID cytidine deaminase to both strands of transcribed duplex DNA substrates. Cell. 2011; 144:353–363. 21255825 10.1016/j.cell.2011.01.001 PMC3065114

[17] Brüning J.G. , Marians K.J. Replisome bypass of transcription complexes and R-loops. Nucleic Acids Res. 2020; 48:10353–10367. 32926139 10.1093/nar/gkaa741 PMC7544221

[18] Kumar C. , Batra S., Griffith J.D., Remus D. The interplay of RNA:DNA hybrid structure and G-quadruplexes determines the outcome of R-loop–replisome collisions. eLife. 2021; 10:e72286. 34494544 10.7554/eLife.72286 PMC8479836

[19] Stoy H. , Zwicky K., Kuster D., Lang K.S., Krietsch J., Crossley M.P., Schmid J.A., Cimprich K.A., Merrikh H., Lopes M. Direct visualization of transcription–replication conflicts reveals post-replicative DNA:RNA hybrids. Nat. Struct. Mol. Biol. 2023; 30:348–359. 36864174 10.1038/s41594-023-00928-6 PMC10023573

[20] Salas M. , Holguera I., Redrejo-Rodríguez M., de Vega M. DNA-binding proteins essential for protein-primed bacteriophage Φ29 DNA replication. Front Mol. Biosci. 2016; 3:37. 27547754 10.3389/fmolb.2016.00037 PMC4974454

[21] Garmendia C. , Bernad A., Esteban J.A., Blanco L., Salas M. The bacteriophage phi 29 DNA polymerase, a proofreading enzyme. J. Biol. Chem. 1992; 267:2594–2599. 1733957

[22] Esteban J.A. , Salas M., Blanco L. Fidelity of phi 29 DNA polymerase. Comparison between protein-primed initiation and DNA polymerization. J. Biol. Chem. 1993; 268:2719–2726. 8428945

[23] Kamtekar S. , Berman A.J., Wang J., Lázaro J.M., de Vega M., Blanco L., Salas M., Steitz T.A. Insights into strand displacement and processivity from the crystal structure of the protein-primed DNA polymerase of bacteriophage phi29. Mol. Cell. 2004; 16:609–618. 15546620 10.1016/j.molcel.2004.10.019

[24] Rodríguez I. , Lázaro J.M., Blanco L., Kamtekar S., Berman A.J., Wang J., Steitz T.A., Salas M., de Vega M. A specific subdomain in phi29 DNA polymerase confers both processivity and strand-displacement capacity. Proc. Natl Acad. Sci. USA. 2005; 102:6407–6412. 15845765 10.1073/pnas.0500597102 PMC1088371

[25] Fuller C.W. , Kumar S., Porel M., Chien M., Bibillo A., Stranges P.B., Dorwart M., Tao C., Li Z., Guo W. et al. Real-time single-molecule electronic DNA sequencing by synthesis using polymer-tagged nucleotides on a nanopore array. Proc. Natl Acad. Sci. USA. 2016; 113:5233–5238. 27091962 10.1073/pnas.1601782113 PMC4868432

[26] Manrao E.A. , Derrington I.M., Laszlo A.H., Langford K.W., Hopper M.K., Gillgren N., Pavlenok M., Niederweis M., Gundlach J.H. Reading DNA at single-nucleotide resolution with a mutant MspA nanopore and phi29 DNA polymerase. Nat. Biotechnol. 2012; 30:349–353. 22446694 10.1038/nbt.2171 PMC3757088

[27] Wang W. , Ren Y., Lu Y., Xu Y., Crosby S.D., Di Bisceglie A.M., Fan X. Template-dependent multiple displacement amplification for profiling human circulating RNA. BioTechniques. 2017; 63:21–27. 28701144 10.2144/000114566 PMC5762180

[28] Gadkar V. , Rillig M.C. Application of Phi29 DNA polymerase mediated whole genome amplification on single spores of arbuscular mycorrhizal (AM) fungi. FEMS Microbiol. Lett. 2005; 242:65–71. 15621421 10.1016/j.femsle.2004.10.041

[29] Cheon N.Y. , Kim H.-S., Yeo J.-E., Schärer O.D., Lee J.Y. Single-molecule visualization reveals the damage search mechanism for the human NER protein XPC-RAD23B. Nucleic Acids Res. 2019; 47:8337–8347. 31372632 10.1093/nar/gkz629 PMC6895271

[30] Kang Y. , Cho C., Lee K.S., Song J.J., Lee J.Y. Single-molecule imaging reveals the mechanism underlying histone loading of *Schizosaccharomyces pombe*AAA+ ATPase Abo1. Mol. Cells. 2021; 44:79–87. 33658433 10.14348/molcells.2021.2242 PMC7941004

[31] Kang Y. , Han Y.G., Khim K.W., Choi W.G., Ju M.K., Park K., Shin K.J., Chae Y.C., Choi J.H., Kim H. et al. Alteration of replication protein A binding mode on single-stranded DNA by NSMF potentiates RPA phosphorylation by ATR kinase. Nucleic Acids Res. 2023; 51:7936–7950. 37378431 10.1093/nar/gkad543 PMC10450186

[32] Kang Y. , Cheon N.Y., Cha J., Kim A., Kim H.I., Lee L., Kim K.O., Jo K., Lee J.Y. High-throughput single-molecule imaging system using nanofabricated trenches and fluorescent DNA-binding proteins. Biotechnol. Bioeng. 2020; 117:1640–1648. 32162675 10.1002/bit.27331

[33] Kang H.J. , Cheon N.Y., Park H., Jeong G.W., Ye B.J., Yoo E.J., Lee J.H., Hur J.H., Lee E.A., Kim H. et al. TonEBP recognizes R-loops and initiates m6A RNA methylation for R-loop resolution. Nucleic Acids Res. 2021; 49:269–284. 33313823 10.1093/nar/gkaa1162 PMC7797050

[34] Ibarra B. , Chemla Y.R., Plyasunov S., Smith S.B., Lázaro J.M., Salas M., Bustamante C. Proofreading dynamics of a processive DNA polymerase. EMBO J. 2009; 28:2794–2802. 19661923 10.1038/emboj.2009.219 PMC2750014

[35] Morin J.A. , Cao F.J., Lázaro J.M., Arias-Gonzalez J.R., Valpuesta J.M., Carrascosa J.L., Salas M., Ibarra B. Active DNA unwinding dynamics during processive DNA replication. Proc. Natl Acad. Sci. USA. 2012; 109:8115–8120. 22573817 10.1073/pnas.1204759109 PMC3361432

[36] Morin J.A. , Cao F.J., Lázaro J.M., Arias-Gonzalez J.R., Valpuesta J.M., Carrascosa J.L., Salas M., Ibarra B. Mechano-chemical kinetics of DNA replication: identification of the translocation step of a replicative DNA polymerase. Nucleic Acids Res. 2015; 43:3643–3652. 25800740 10.1093/nar/gkv204 PMC4402526

[37] Blanco L. , Bernad A., Lázaro J.M., Martín G., Garmendia C., Salas M. Highly efficient DNA synthesis by the phage phi 29 DNA polymerase. Symmetrical mode of DNA replication. J. Biol. Chem. 1989; 264:8935–8940. 2498321

[38] Vlček Č. , Pačes V. Nucleotide sequence of the late region of Bacillus phage φ29 completes the 19285-bp sequence of φ29 genome. Comparison with the homologous sequence of phage PZA. Gene. 1986; 46:215–225. 3803926 10.1016/0378-1119(86)90406-3

[39] Schauer G.D. , Spenkelink L.M., Lewis J.S., Yurieva O., Mueller S.H., van Oijen A.M., O’Donnell M.E Replisome bypass of a protein-based R-loop block by Pif1. Proc. Natl Acad. Sci. USA. 2020; 117:30354–30361. 33199603 10.1073/pnas.2020189117 PMC7720201

[40] Roberts R.W. , Crothers D.M. Stability and properties of double and triple helices: dramatic effects of RNA or DNA backbone composition. Science. 1992; 258:1463–1466. 1279808 10.1126/science.1279808

[41] Kraeva R.I. , Krastev D.B., Roguev A., Ivanova A., Nedelcheva-Veleva M.N., Stoynov S.S. Stability of mRNA/DNA and DNA/DNA duplexes affects mRNA transcription. PLoS One. 2007; 2:e290. 17356699 10.1371/journal.pone.0000290 PMC1808433

[42] Kumar C. , Batra S., Griffith J.D., Remus D. The interplay of RNA:DNA hybrid structure and G-quadruplexes determines the outcome of R-loop–replisome collisions. eLife. 2021; 10:e72286. 34494544 10.7554/eLife.72286 PMC8479836

[43] Lim G. , Hohng S. Single-molecule fluorescence studies on cotranscriptional G-quadruplex formation coupled with R-loop formation. Nucleic Acids Res. 2020; 48:9195–9203. 32810236 10.1093/nar/gkaa695 PMC7498336

[44] Lee C.-Y. , McNerney C., Ma K., Zhao W., Wang A., Myong S. R-loop induced G-quadruplex in non-template promotes transcription by successive R-loop formation. Nat. Commun. 2020; 11:3392. 32636376 10.1038/s41467-020-17176-7 PMC7341879

[45] De Magis A. , Manzo S.G., Russo M., Marinello J., Morigi R., Sordet O., Capranico G. DNA damage and genome instability by G-quadruplex ligands are mediated by R loops in human cancer cells. Proc. Natl Acad. Sci. USA. 2019; 116:816–825. 30591567 10.1073/pnas.1810409116 PMC6338839

[46] Duquette M.L. , Handa P., Vincent J.A., Taylor A.F., Maizels N. Intracellular transcription of G-rich DNAs induces formation of G-loops, novel structures containing G4 DNA. Genes Dev. 2004; 18:1618–1629. 15231739 10.1101/gad.1200804 PMC443523

[47] Nagesh N. , Chatterji D. Ammonium ion at low concentration stabilizes the G-quadruplex formation by telomeric sequence. J Biochem. Biophys. Methods. 1995; 30:1–8. 7608467 10.1016/0165-022x(94)00057-k

[48] Olejko L. , Dutta A., Shahsavar K., Bald I. Influence of different salts on the G-quadruplex structure formed from the reversed human telomeric DNA sequence. Int. J. Mol. Sci. 2022; 23:12206. 36293060 10.3390/ijms232012206 PMC9602856

[49] D’Atri V. , Gabelica V. DNA and RNA telomeric G-quadruplexes: what topology features can be inferred from ion mobility mass spectrometry?. Analyst. 2019; 144:6074–6088. 31528871 10.1039/c9an01216h

[50] Kosman J. , Juskowiak B. Hemin/G-quadruplex structure and activity alteration induced by magnesium cations. Int. J. Biol. Macromol. 2016; 85:555–564. 26778160 10.1016/j.ijbiomac.2016.01.020

[51] Ambrus A. , Chen D., Dai J., Bialis T., Jones R.A., Yang D. Human telomeric sequence forms a hybrid-type intramolecular G-quadruplex structure with mixed parallel/antiparallel strands in potassium solution. Nucleic Acids Res. 2006; 34:2723–2735. 16714449 10.1093/nar/gkl348 PMC1464114

[52] Dai J. , Carver M., Punchihewa C., Jones R.A., Yang D. Structure of the hybrid-2 type intramolecular human telomeric G-quadruplex in K+ solution: insights into structure polymorphism of the human telomeric sequence. Nucleic Acids Res. 2007; 35:4927–4940. 17626043 10.1093/nar/gkm522 PMC1976458

[53] Wang W. , Hu S., Gu Y., Yan Y., Stovall D.B., Li D., Sui G. Human MYC G-quadruplex: from discovery to a cancer therapeutic target. Biochim. Biophys. Acta Rev. Cancer. 2020; 1874:188410. 32827579 10.1016/j.bbcan.2020.188410

[54] Dickerhoff J. , Dai J., Yang D. Structural recognition of the MYC promoter G-quadruplex by a quinoline derivative: insights into molecular targeting of parallel G-quadruplexes. Nucleic Acids Res. 2021; 49:5905–5915. 33978746 10.1093/nar/gkab330 PMC8191789

[55] You H. , Wu J., Shao F., Yan J. Stability and kinetics of c-MYC promoter G-quadruplexes studied by single-molecule manipulation. J. Am. Chem. Soc. 2015; 137:2424–2427. 25654467 10.1021/ja511680u

[56] Yang D. , Hurley L.H. Structure of the biologically relevant G-quadruplex in the c-MYC promoter. Nucleosides Nucleotides Nucleic Acids. 2006; 25:951–968. 16901825 10.1080/15257770600809913

[57] Wang Y.R. , Guo T.T., Zheng Y.T., Lai C.W., Sun B., Xi X.G., Hou X.M. Replication protein A plays multifaceted roles complementary to specialized helicases in processing G-quadruplex DNA. iScience. 2021; 24:102493. 34113828 10.1016/j.isci.2021.102493 PMC8169993

[58] Woodford K.J. , Usdin K., Weitzmann M.N. DNA secondary structures and the evolution of hypervariable tandem arrays. J. Biol. Chem. 1997; 272:9517–9523. 9083093 10.1074/jbc.272.14.9517

[59] Sarkies P. , Reams C., Simpson L.J., Sale J.E. Epigenetic instability due to defective replication of structured DNA. Mol. Cell. 2010; 40:703–713. 21145480 10.1016/j.molcel.2010.11.009 PMC3145961

[60] Olsen C.M. , Gmeiner W.H., Marky L.A. Unfolding of G-quadruplexes: energetic, and ion and water contributions of G-quartet stacking. J. Phys. Chem. B. 2006; 110:6962–6969. 16571009 10.1021/jp0574697

[61] Lane A.N. , Chaires J.B., Gray R.D., Trent J.O. Stability and kinetics of G-quadruplex structures. Nucleic Acids Res. 2008; 36:5482–5515. 18718931 10.1093/nar/gkn517 PMC2553573

[62] Soengas M.S. , Esteban J.A., Lázaro J.M., Bernad A., Blasco M.A., Salas M., Blanco L. Site-directed mutagenesis at the exo III motif of phi 29 DNA polymerase; overlapping structural domains for the 3'–5' exonuclease and strand-displacement activities. EMBO J. 1992; 11:4227–4237. 1396603 10.1002/j.1460-2075.1992.tb05517.x PMC556934

[63] de Vega M. , Lazaro J.M., Salas M., Blanco L. Primer-terminus stabilization at the 3'–5' exonuclease active site of phi29 DNA polymerase. Involvement of two amino acid residues highly conserved in proofreading DNA polymerases. EMBO J. 1996; 15:1182–1192. 8605889 PMC450017

[64] Manthei K.A. , Hill M.C., Burke J.E., Butcher S.E., Keck J.L. Structural mechanisms of DNA binding and unwinding in bacterial RecQ helicases. Proc. Natl Acad. Sci. USA. 2015; 112:4292–4297. 25831501 10.1073/pnas.1416746112 PMC4394303

[65] Velankar S.S. , Soultanas P., Dillingham M.S., Subramanya H.S., Wigley D.B. Crystal structures of complexes of PcrA DNA helicase with a DNA substrate indicate an inchworm mechanism. Cell. 1999; 97:75–84. 10199404 10.1016/s0092-8674(00)80716-3

[66] Pomerantz R.T. , O’Donnell M. Direct restart of a replication fork stalled by a head-on RNA polymerase. Science. 2010; 327:590–592. 20110508 10.1126/science.1179595 PMC2861996

[67] Deshpande A.M. , Newlon C.S. DNA replication fork pause sites dependent on transcription. Science. 1996; 272:1030–1033. 8638128 10.1126/science.272.5264.1030

[68] Miglietta G. , Russo M., Capranico G. G-quadruplex–R-loop interactions and the mechanism of anticancer G-quadruplex binders. Nucleic Acids Res. 2020; 48:11942–11957. 33137181 10.1093/nar/gkaa944 PMC7708042

[69] Graf M. , Bonetti D., Lockhart A., Serhal K., Kellner V., Maicher A., Jolivet P., Teixeira M.T., Luke B. Telomere length determines TERRA and R-loop regulation through the cell cycle. Cell. 2017; 170:72–85. 28666126 10.1016/j.cell.2017.06.006

[70] Fernandes R.V. , Feretzaki M., Lingner J. The makings of TERRA R-loops at chromosome ends. Cell Cycle. 2021; 20:1745–1759. 34432566 10.1080/15384101.2021.1962638 PMC8525998

[71] Balk B. , Maicher A., Dees M., Klermund J., Luke-Glaser S., Bender K., Luke B. Telomeric RNA–DNA hybrids affect telomere-length dynamics and senescence. Nat. Struct. Mol. Biol. 2013; 20:1199–1205. 24013207 10.1038/nsmb.2662

[72] Chaires J.B. Human telomeric G-quadruplex: thermodynamic and kinetic studies of telomeric quadruplex stability. FEBS J. 2010; 277:1098–1106. 19951355 10.1111/j.1742-4658.2009.07462.x PMC2896281

