# Supplementary material for: Direct visualization of replication and R-loop collision using single-molecule imaging
Source: Nucleic Acids Res. 2023 Nov 22;52(1):259–73. doi: 10.1093/nar/gkad1101 (PMC10783495; doi:10.1093/nar/gkad1101)
Supplement: gkad1101_Supplemental_Files [file gkad1101_supplemental_files.zip › RevisedSupplementaryInformation_NAR-02103-J-2023.docx]

**Direct visualization of replication and R-loop collision using single-molecule imaging**

Subin Kim^1^, Woo Hee Shin^1^, Yujin Kang^1^, Hongtae Kim^1^, and Ja Yil Lee^1,2*^

^1^ Department of Biological Sciences, Ulsan National Institute of Science and Technology, Ulsan, 44919, Republic of Korea

^2^ Institute of Basic Science Center for Genomic Integrity, Ulsan, 44919, Republic of Korea

* Corresponding author: Ja Yil Lee: [biojayil@unist.ac.kr](mailto:biojayil@unist.ac.kr)

**Contents**

**I. Supplementary Materials and Methods**

**1. DNA preparation**

1-1. Preparation of engineered lambda DNA (λ-I3)

1-2. R-loop, D-loop, or bubble insertion into λ-I3

1-3. T7 promoter insertion into lambda DNA(λ-DNA)

**2. Protein purification and activity test**

2-1. Purification of Phi29 DNA polymerase (Phi29 DNAp)

2-2. Rolling circle replication assay with purified Phi29 DNAp

2-3. Purification of human RPA tagged with enhanced green fluorescent protein

(RPA-eGFP)

2-4. Electrophoretic mobility shift assay with RPA-eGFP

2-5. Purification of *E. coli* single-stranded DNA binding protein (EcSSB)

**3. *In vitro* T7 RNA polymerase transcription assay**

**4. DNA curtain assay and data analyses**

4-1. Preparation of DNA curtain assay

4-2. Phi29 DNAp replication

4-3. T7 RNAp transcription

4-4. Collision experiment between Phi29 DNAp and T7 RNA transcripts

4-5. Collision experiment between Phi29 DNAp and T7 RNAp bound to T7 promoter

4-6. Data analyses

**5. Circular dichroism assay**

**II. Supplementary Figures**

Figure S1. Purification of Phi29 DNAp and RPA-eGFP and their activity test

Figure S2. Effect of *E. coli* SSB on the replication of Phi29 DNAp

Figure S3. Preparation of single R-loop containing lambda DNA (λ-I3)

Figure S4. Collision of Phi29 DNAp with RPA-bound bubble, R-loop, or D-loop

Figure S5. The fate of 20-nt RNA at the R-loop after the collision of Phi29 DNAp with the R-loop

Figure S6. Pass and stall fractions depending on RNA constructs at the R-loop

Figure S7. Temperature-dependent circular dichroism for RNA-DNA hybrid and DNA duplex

Figure S8. *In vitro* G-quadruplex formation

Figure S9. *In vitro* transcription of T7 RNAp

**III. Supplementary Table**

**IV. References**

**I. Supplementary Materials and Methods**

**1. DNA preparation**

All oligomers were synthesized from Bioneer (South Korea), Bionics (South Korea), or IDT Technology (USA) and listed in Supplementary Table.

*1-1. Preparation of engineered lambda DNA (λ-I3)*

λ-I3 (47,472 bp) is an engineered lambda phage DNA (λ-DNA) that contains seven nickase sites (Nt.BspQI) between 33,514 bp and 33,630 bp (Supplementary Figure S3). λ-I3 was packaged into lambda phage particles using MaxPlax λ extracts (MP5105, Epicentre). The phage particles were then infected into *E. coli* LE302MP strain. The infected *E. coli* cells were incubated at 37°C for 20 min and then spread on an agar plate with 0.7% of top agar. After overnight incubation at 37°C, a single plaque was inserted into 800 µl of LE392MP cells, which were prepared by mixing 400 µl of overnight-cultured uninfected LE392MP cells with 400 µl of 10 mM MgCl_2_ and 10 mM CaCl_2_. The mixture with the single plaque was further grown up in 200 ml of NZCYM broth at 37°C with 125 rpm. OD_600_ increased over 1.6 and suddenly dropped down close to ~0.3 because of bacteriophage burst. At that time, 5 ml of chloroform was added to the cells followed by further incubation at 37°C for 15 min. NaCl powder was dissolved to the cells up to 1 M, which were placed on ice for 10 min. The cells were pelleted at 12,000 g for 10 min and the supernatant was collected. PEG20000 (81300, Sigma) was added to the supernatant up to 10% (w/v) and incubated at 4°C at least overnight. The phage particles were harvested by centrifugation at 12,000 g for 15 min. The phage pellets were resuspended in SM buffer (10 mM Tris-HCl [8.3], 100 mM NaCl, and 10 mM MgCl_2_), and then RNase A (R4875-500, Sigma) and DNase I (D5319-2, Sigma) were added to remove DNA and RNA, respectively. Proteinase K (P2308, Sigma) was treated and incubated at 37°C for 45 min to eliminate RNase A and DNase I. Proteinase K was then inactivated by incubation at 65°C for 10 min with 1.25% of SDS and 100 mM EDTA. Lambda DNA was obtained by isopropanol precipitation.

*1-2. R-loop, D-loop, or bubble insertion into λ-I3*

To insert a single R-loop or D-loop into λ-I3, bubble construct was first formed on λ-I3 (Supplementary Figure S3A). Nt.BspQI (6 units/ml) (R0644S, NEB) was treated to 7 nM of λ-I3 in 1x NEBuffer r3.1 (100 mM NaCl, 50 mM Tris-HCl [7.9], 10 mM MgCl_2_, 100 μg/ml recombinant albumin) (B6003S, NEB) with 1 mM ATP at 50℃ for 1 hr. Proteinase K was treated at 50℃ for 1 hr to inactivate the nickase. 1 μM of Lambda R-biotin (or Lambda L-biotin), 1 μM of Lambda L (Lambda R), 2 μM of Phi29 Primer, and 3.5 μM of Bubble Oligo were incubated with 10 nM of the nicked λ-I3 at 80°C for 20 min and slowly cooled down to room temperature (25°C) (Supplementary Table). During this process, the region between 33,514 bp and 33,630 bp was denatured out to form a gap and then annealed with the Bubble oligomer to form a large bubble structure, and either end of the λ-I3 had biotin or a primer. The residual short oligomer fragments from the nickase sites were removed by MicroSpin S-400 HR Columns (27514001, Cytiva). All nicks were then sealed by T4 DNA ligase (M001S, Enzynomics) at room temperature overnight. 5 μM of RNA or DNA complementary to the bubble was annealed with 5 nM of the bubble-containing λ-I3 by heating at 65°C for 20 min followed by slow cooling to room temperature. Excessive RNA or DNA oligomers were removed by MicroSpin S-400 HR Columns (27514001, Cytiva).

*1-3. T7 promoter insertion into lambda DNA (λ-DNA)*

For the transcription of T7 RNA polymerase (T7 RNAp), T7 Promoter and T7 Promoter_comp were annealed and inserted between XhoI and NheI sites of λ-DNA (N3011, NEB) (Supplementary Table). λ-DNA was digested with XhoI and NheI, and the annealed DNA containing T7 promoter was ligated with the digested λ-DNA at 100:1 molar ratio. The ligated λ-DNA was then amplified by lambda packaging using MaxPlax™ Lambda Packaging Extracts (MP5105, Epicentre) with the same protocol as described above. For DNA curtain assays, 10 nM of T7 promoter containing λ-DNA was annealed with 1 μM of Lambda R-biotin (or Lambda L-biotin), 1 uM of Lambda L (Lambda R) and 2 μM of Phi29 Primer at 65°C for 20 min followed by slow cooling down to room temperature. All nicks were sealed by T4 DNA ligase (M001, Enzynomics), which was then heat-inactivated. Excessive oligomers were removed by Microspin S-400 HR Columns (27514001, Cytiva).

**2. Protein purification and activity test**

All protein purification procedures were conducted at 4℃.

*2-1. Purification of Phi29 DNA polymerase (Phi29 DNAp)*

For Phi29 DNAp purification, we followed a previous protocol with slight modifications (1). Phi29 DNAp gene was subcloned into a pTXB1-derived plasmid containing 3xFLAG and 6x histidine residues (His) at the amino (N)-terminus and chitin-binding domain at the carboxyl (C)-terminus. The plasmid was transformed into *E. coli* BL21(DE3) strain (CP110, Enzynomics). Cells were grown in 2 L LB broth at 37℃ until OD_600_ reached about 0.6. Proteins were expressed by 1 mM IPTG (Isopropyl β-D-1-thiogalactopyranoside) at 16℃ overnight. The cells were harvested at 8,000 g for 10 min and resuspended in resuspension buffer (25 mM Tris-HCl [8.0], 500 mM NaCl, 5% glycerol, and 5 mM imidazole) with 1x protease inhibitor (Halt, 78439, Thermo Fisher Scientific). The cells were then lysed by sonication and then pelleted by ultracentrifugation at 90,000 g for 40 min. Clarified lysates were filtered with 0.22 μm syringe filters (J1.F204.030N, Biofil) and loaded onto a gravity column with 10 ml of Ni-NTA resin (HisPur™ Ni-NTA resin, 88222, Thermo Fisher Scientific) equilibrated with 50 ml of Ni-wash buffer (25 mM Tris-HCl [8.0], 500 mM NaCl, 5% glycerol, and 5 mM imidazole). The Ni-NTA resin was washed with 50 ml of Ni-wash buffer, and proteins were eluted by Ni-elution buffer (25 mM Tris-HCl [8.0], 500 mM NaCl, 5% glycerol, and 300 mM imidazole). The eluates were then loaded onto 5 ml of chitin-resin in a gravity column (S6651S, New England Biolabs) equilibrated with chitin-wash buffer (25 mM Tris-HCl [7.4], 500 mM NaCl, and 0.1 mM EDTA). The resin was washed with 25 ml of chitin-wash buffer and incubated overnight with chitin-elution buffer (25 mM Tris-HCl [7.4], 500 mM NaCl, 0.1 mM EDTA, and 50 mM DTT), which cleaved chitin binding domain. Eluates containing Phi29 DNAp were pooled and dialyzed against Phi29 dialysis buffer (10 mM Tris-HCl [7.4], 100 mM KCl, 1 mM DTT, 0.1 mM EDTA, and 50% glycerol) overnight and stored at -80℃.

*2-2. Rolling circle replication assay with purified Phi29 DNAp*

40 nM of M13mp18 ssDNA (N4040S, New England Biolabs) was annealed with 33.3 nM of RCR primer in annealing buffer (40 mM Tris-HCl [8.0], 50 mM NaCl, and 10 mM MgCl_2_) (Supplementary Table). Then 8 nM of the annealed M13mp18 ssDNA was mixed with 46.3 nM of purified Phi29 DNAp or a commercial one (M0269, NEB) in Phi29 DNAp buffer (50 mM Tris-HCl [7.5], 10 mM MgCl_2_, 10 mM (NH_4_)_2_SO_4_, and 4 mM DTT (DL-dithiothreitol) supplemented with 1 mM dNTPs. The reactants were incubated at 30℃ for 10 or 40 min and then deproteinized with 1 mg/ml protease K at 37℃ for 20 min. Then the reactants were analyzed by 0.8% alkaline agarose gel electrophoresis with SYBR Gold staining (S11494, Invitrogen).

*2-3. Purification of human RPA with enhanced green fluorescent protein (RPA-eGFP)*

RPA labeled with enhanced green fluorescent protein (RPA-eGFP) purification was conducted by following a previous protocol (2). RPA-eGFP gene was subcloned into a pET11c-drived plasmid containing 8xHis at the C-terminus. RPA-eGFP was expressed in *E. coli* BL21(DE3) strain. Cells were grown in 6 L LB broth at 37℃. At OD_600_ ~0.6, 1 mM IPTG was treated to induce protein expression, and the proteins were over-expressed at 16℃ overnight. Cells were harvested at 8,000 g for 10 min and resuspended in T-100 buffer (25 mM Tris-HCl [7.5], 100 mM NaCl, 0.5 mM EDTA, 1 mM DTT, and 10% glycerol) with 1x protease inhibitor (Halt, 78439, Thermo Fisher Scientific). The cells were lysed by sonication and centrifuged at 100,000 g for 1 hr. Clarified lysates were filtered using 0.22 μm syringe filters and then loaded onto 20 ml of affi-gel blue column (1537301, Biorad), which were washed with 100 ml of T-100 buffer and then equilibrated with 100 ml of T-300 buffer (25 mM Tris-HCl [7.5], 300 mM NaCl, 0.5 mM EDTA, 1 mM DTT, and 10% glycerol). The proteins were eluted by salt gradient from T-300 buffer to T-2500 NaSCN buffer (25 mM Tris-HCl [7.5], 2.5 M NaSCN, 0.5 mM EDTA, 1 mM DTT, and 10% glycerol). Eluates were then loaded onto a gravity-flow column with 15 ml Talon resin (635502, Takara) equilibrated with Talon wash buffer (50 mM HEPES-NaOH [7.5], 250 mM NaCl, and 20 mM imidazole). The Talon resin was washed again with 75 ml of Talon wash buffer, and the proteins were eluted with Talon elution buffer (50 mM HEPES-NaOH [7.5], 250 mM NaCl, and 250 mM imidazole). Eluates were dialyzed against RPA dialysis buffer (50 mM Tris-HCl [8.0], 40 mM NaCl, 1 mM DTT, and 0.5 mM EDTA) and stored at -80°C.

*2-4. Electrophoretic mobility shift assay with RPA-eGFP*

10 nM of Cy5-lableled 91-nt ssDNA was mixed with RPA-eGFP at different concentrations (0, 10, 25, 50, 100, and 150 nM) in 1x RPA-eGFP reaction buffer (50 mM Tris-HCl [7.5], 50 mM NaCl, and 4 mM DTT) and incubated at room temperature for 20 min. Reactants were analyzed by 5% nondenaturing polyacrylamide gel electrophoresis in 1x TBE buffer at 4℃.

To test RPA-eGFP binding to GQ, 10 nM of 1x hTel_GQ (d(GGGTTA)_4_) labeled with Cy5 was incubated with RPA-eGFP at different concentrations (0, 1, 3, 10, and 30 nM) in Phi29 DNAp buffer supplemented with 50 mM KCl. Then reactants were analyzed by 6% nondenaturing polyacrylamide gel electrophoresis in 1x TBE buffer supplemented with 50 mM KCl at 4℃.

*2-5. Purification of E. coli single-stranded DNA binding protein (EcSSB)*

*Ec*SSB gene with eGFP at C-terminus was subcloned to into a pGV358s2-derived plasmid that contains Streptag at C-terminus. The plasmid was transformed to *E. coli* BL21(DE3), and cells were grown until OD_600_ became ~0.6 in 6 L LB broth at 37°C. 1 mM IPTG was added to the cells for protein expression, and the cells were further grown at 16°C overnight. Cells were pelleted by centrifugation at 8,000 g for 10 min, and the cell pellets were resuspended in SSB resuspension buffer (25 mM Tris-HCl [7.4], 500 mM NaCl, 5% (v/v) glycerol, 1 mM EDTA, and 1 mM PMSF) supplemented with 1x protease inhibitor (Halt, 78439, Thermo Fisher Scientific). The cells were lysed by sonication and centrifuged at 40,000 g for 45 min. Clarified lysates were filtered with 0.22 µm syringe filters and loaded onto a 10 ml of Strep-Tactin XT 4 gravity-flow column (2-5010-025, IBA Lifesciences) equilibrated with SSB resuspension buffer. The column was washed with 50 ml of SSB resuspension buffer and incubated with SSB elution buffer (25 mM Tris-HCl [7.4], 500 mM NaCl, 5% (v/v) glycerol, 1 mM EDTA, 1 mM PMSF, and 50 mM biotin) overnight. Eluted proteins were dialyzed against SSB dialysis buffer (50 mM Tris-HCl [7.4], 300 mM NaCl, and 50% glycerol) and stored at -80°C.

**3. *In vitro* T7 RNA polymerase transcription assay**

For *in vitro* transcription of T7 RNAp, we performed PCR to amplify 2,051 bp DNA fragment containing T7 promoter at 418 bp from one end. 20 nM of the T7 promoter-containing DNA fragment was mixed with 0.5 mM rNTPs, Cy5-UTP (B8333, APExBIO) at different concentrations, and T7 RNAp at various amount in RNAp buffer (40 mM Tris-HCl [8.8], 6 mM MgCl_2_, 1 mM DTT, and 2 mM spermidine (124-20-9, Sigma)), which was slightly modified from previous protocols (3,4). The reactants were incubated at 37°C for 1 hr. Then, 0.05 mg/ml of DNase I (D5319-2, Sigma) was added and incubated at 37°C for 10 min. The reaction was stopped by adding stopping buffer (95% formamide and 20 mM EDTA) with equal volume to the reaction volume and successively by heating the reactants at 95°C for 2 min. The transcripts were analyzed by 7% denaturing polyacrylamide gel with 7 M urea in 1x TBE buffer. Cy5 fluorescence was imaged by Typhoon RGB (Cytiva).

For the binding of T7 RNAp to the T7 promoter site, we performed EMSA. 20 nM of T7 Promoter_EMSA and T7 Promoter_EMSA_comp oligomers were hybridized (Supplementary Table). The DNA construct was mixed with different concentrations (0, 10, 20, 100, and 200 nM) of T7 RNAp in RNAp buffer without rNTPs and incubated at room temperature for 5 min. Reactants were analyzed by 6% nondenaturing polyacrylamide gel electrophoresis in 1x TBE buffer at 4℃.

**4. DNA curtain assay and data analyses**

*4-1. Preparation of DNA curtain assay*

All DNA curtain experiments were conducted at room temperature. Fused-silica slides that had two holes and nano-trench patterns were glued with coverslips via double-sided tape to form microchamber-containing flowcells (5). Liposomes composed of 0.5% biotinylated-DOPE (1,2-dioleoyl-sn-glycero-3-phosphoethanolamine-N-(cap biotinyl) (sodium salt), 870273, Avanti Polar Lipids), 8% mPEG 2000-DOPE (1,2-dioleoyl-sn-glycero-3-phosphoethanolamine-N-[methoxy(polyethylene glycol)-2000], 880130, Avanti Polar Lipids) and DOPC (1,2-dioleoyl-sn-glycero-3-phophocholine, 850375, Avanti Polar Lipids) were incubated to flowcells to deposit lipid bilayer on the slide surface. The flowcell surface was further passivated with 0.4% BSA (bovine serum albumin, A7030, Sigma) and then 25 μg/ml of streptavidin was injected.

*4-2. Phi29 DNAp replication*

For Phi29 DNAp replication, 2 nM of λ-DNA was pre-incubated with 240 nM of Phi29 DNAp at 30°C for 10 min in Phi29 DNAp buffer (50 mM Tris-HCl [7.5], 10 mM (NH_4_)_2_SO_4_, 10 mM MgCl_2_, and 4 mM DTT supplemented with 0.1 mM dNTP without dGTP to attach Phi29 DNAp at the primer. Then the pre-incubated λ-DNA was diluted in 400 ul of imaging buffer (50 mM Tris-HCl [8.8], 10 mM (NH_4_)_2_SO_4_, 10 mM MgCl_2_, 4 mM DTT, 3.2% glucose, and 0.1x gloxy) and anchored to the lipid bilayer via biotin-streptavidin linkage. DNA curtains were formed in imaging buffer. Replication reaction was started in the curtains by switching imaging buffer to replication buffer (imaging buffer supplemented with 3 nM RPA-eGFP and dNTP (0.1 mM unless specified) at a flow rate of 0.5 ml/min. As a control for the bubble construct, 2 nM of λ-I3 containing an R-loop was pre-incubated with 1 ul of RNase H (M0297L, NEB) in RNase H reaction buffer (B0297L, NEB) for 10 min at 37℃ to eliminate RNA from the R-loop. Then DNA curtain assay was performed as above. For Q-quadruplex formation in an R-loop, 50 mM KCl was added to imaging buffer and replication buffer. RPA-eGFP and Cy5-labeled RNA were excited by 488 nm and 637 nm lasers (Coherent), respectively, using prism-type total internal reflection fluorescence microscopy (TIRFM) (6,7). The fluorescence signal was imaged by NIS-Element software (Nikon) with 0.1 sec exposure and 0.4 sec shuttering (0.5 sec/frame) for 6 min. For the replication resumption after R-loop elimination, 83 units/ml of RNase H (M0297L, NEB) was injected into the DNA curtain in replication buffer supplemented with 75 mM KCl to remove RNA. To test whether or not RNA remained at the R-loop when Phi29 DNAp was stalled, 637 nm laser was switched on for Cy5 fluorescence imaging immediately after 488 nm laser for replication was turned off.

*4-3. T7 RNAp transcription*

2 nM of T7 promoter-containing λ-DNA was pre-incubated with 0.5 mM rNTPs, 2.5 µM Cy5-UTP (B8333, APExBIO), and 1 µl of T7 RNAp (M0251, NEB) in RNAp buffer for 5 min. Reactants were diluted in 200 µl of RNAp buffer and anchored on the lipid bilayer in the a flowcell. To eliminate T7 RNAp from RNA transcripts on the λ-DNA, λ-DNA was heated at 75°C based on manufacturer’s protocol (NEB) or treated by proteinase K, which was then heat-inactivated at 55°C and filtered by MicroSpin S-400 HR Columns (27514001, Cytiva). Then the transcripts were fluorescently imaged in RNAp buffer supplemented with 3.2% glucose and 0.1x gloxy under the illumination of 637 nm laser using DNA curtain assay.

*4-4. Collision experiment between Phi29 DNAp and T7 RNA transcripts*

2 nM of λ-DNA with RNA transcripts was incubated with 240 nM Phi29 DNAp and 0.1 mM dNTPs without dGTP in RNAp buffer for 5 min at room temperature. Reactants were diluted in 200 ul of RNAp buffer and then injected into the flowcell. RNAp buffer supplemented with 3.2 % glucose and 0.1x gloxy was used to build up DNA curtains at 0.5 ml/min flow rate. For the collision with Phi29 DNAp, 1 nM eGFP-RPA and 0.1 mM dNTPs were further added to the buffer. Fluorescence signal was imaged with 0.1 sec exposure and 0.4 sec shuttering (0.5 sec/frame) for 6 min.

*4-5. Collision experiment between Phi29 DNAp and T7 RNAp bound to T7 promoter*

For the collision between Phi29 DNAp and T7 RNAp bound to the T7 promoter using DNA curtain assays, 3 nM of λ-DNA containing the T7 promoter was pre-incubated with 2 ul of T7 RNAp, 240 nM of Phi29 DNAp, and 0,1 mM dNTP except for dGTP at room temperature for 10 min in RNAp buffer. Reactants were diluted in 400 ul of RNAp buffer and injected into flow cell. Replication reaction was processed in RNAp buffer with 3.2% glucose and 0.1x gloxy. DNA curtain assays were conducted as above.

*4-6. Data analyses*

All imaging data were exported into TIFF format and analyzed by ImageJ software (NIH). Kymographs for individual replication events were made. Speed was estimated by measuring the slope of replication initiation point to end point. Processivity was estimated by measuring the vertical distance between initiation and end points. Binding position distributions were analyzed by a Plug-In, GaussFit OnSpot (NIH).

**5. Circular dichroism assay**

*In vitro* G-quadruplex formation was measured using a spectropolarimeter (Jasco J-815). 10 µM of 1x hTel_GQ, 2x hTel_GQ, or cMyc GQ oligomer was prepared in Phi29 DNAp buffer or Phi29 DNAp buffer supplemented with 50 mM KCl. Wavelengths from 220 nm to 300 nm were scanned at 50 nm/min. Ellipticity was averaged in triplicate.

For the melting temperature measurement using circular dichroism, Nontemplate R-loop and CD_DNA were hybridized with CD_DNA_comp in Phi29 DNAp buffer to make RNA-DNA and DNA-DNA hybrids, respectively. The temperature of the spectropolarimeter was raised from 25℃ to 98℃ at 2℃/min rate, and spectra between 220 nm to 300 nm were measured every 5℃.

**II. Supplementary Figures**

**Figure S1. Purification of Phi29 DNAp and RPA-eGFP and their activity test**


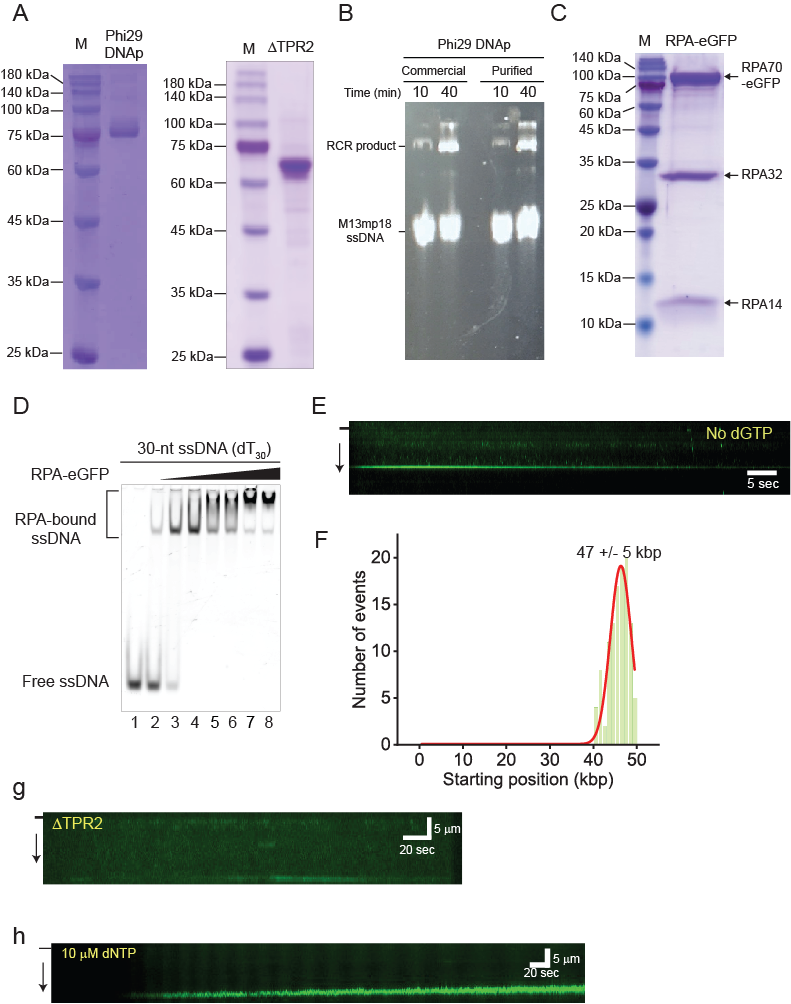


A. SDS PAGE for purified Phi29 DNAp (left) and Phi29 DNAp TPR2 deletion mutant (ΔTPR2) (right).

B. Rolling circle replication to test the replication activity of purified Phi29 DNAp. As a control, we used commercial Phi29 DNAp (M0269, NEB).

C. SDS PAGE for purified human RPA-eGFP, in which eGFP is tagged at the C-terminus of RPA70 subunit.

D. Electrophoretic mobility shift assay for purified RPA-eGFP with 30-nt ssDNA (dT_30_).

E. Kymograph for Phi29 DNAp replication without dGTP in dNTPs. There is no line growth. The black bar and black arrow at the left represent the barrier and flow direction, respectively.

F. Distribution of the replication initiation positions. The distribution is fitted with a single Gaussian function. The fitted center is 47 ± 5 kbp, which is comparable with the length of λ-I3.

G. Kymograph for the replication of ΔTPR2, which exhibits no replication activity. The black bar and black arrow at the left represent the barrier and flow direction, respectively.

H. Kymograph for Phi29 DNAp replication at 10 μM dNTP. The black bar and arrow at the left represent the barrier and flow direction, respectively.

**Figure S2. Effect of *E. coli* SSB on the replication of Phi29 DNAp**


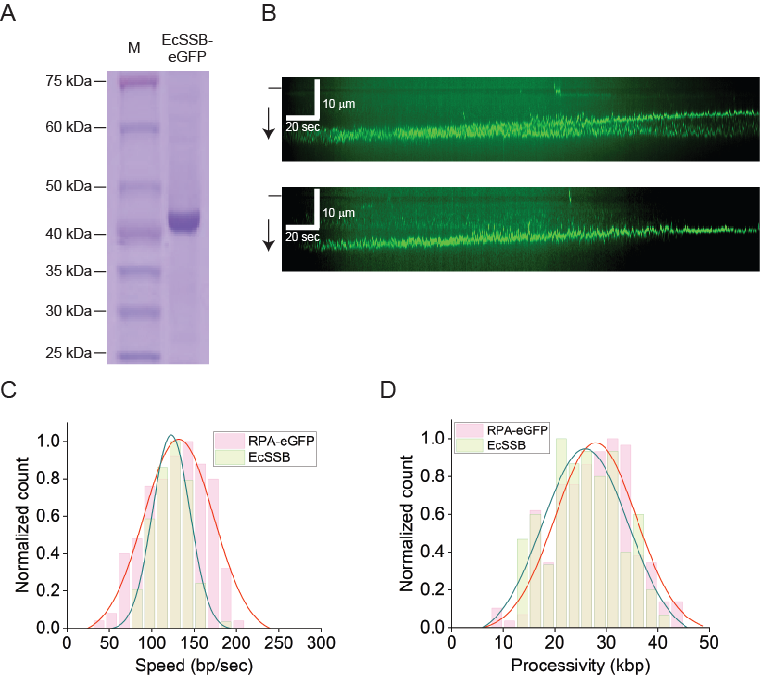


A. SDS PAGE for purified *E. coli* SSB (EcSSB)-eGFP, in which eGFP is tagged at the C-terminus of EcSSB.

B. Kymographs for area (top) and line (bottom) profiles of Phi29 DNAp replication in the presence of EcSSB-eGFP. The black bar and black arrow at the left indicate the barrier and flow direction, respectively.

C. Normalized speed distribution of Phi29 DNAp replication in the presence of RPA-eGFP (magenta) and EcSSB-eGFP (green). Each distribution is fitted with a single Gaussian function. The peak center of RPA-eGFP and EcSSB is 132 ± 48 bp/sec and 123 ± 26 bp/sec, respectively. The number of molecules analyzed are greater than 100.

D. Normalized processivity distributions of Phi29 DNAp in the presence of RPA-eGFP (magenta) and EcSSB-eGFP (green). Each distribution is fitted with a single Gaussian function. The peak center of RPA-eGFP and EcSSB is 28 ± 8 kbp and 26 ± 9 kbp, respectively. The number of molecules analyzed are greater than 100.

**Figure S3. Preparation of single R-loop containing lambda DNA (λ-I3)**


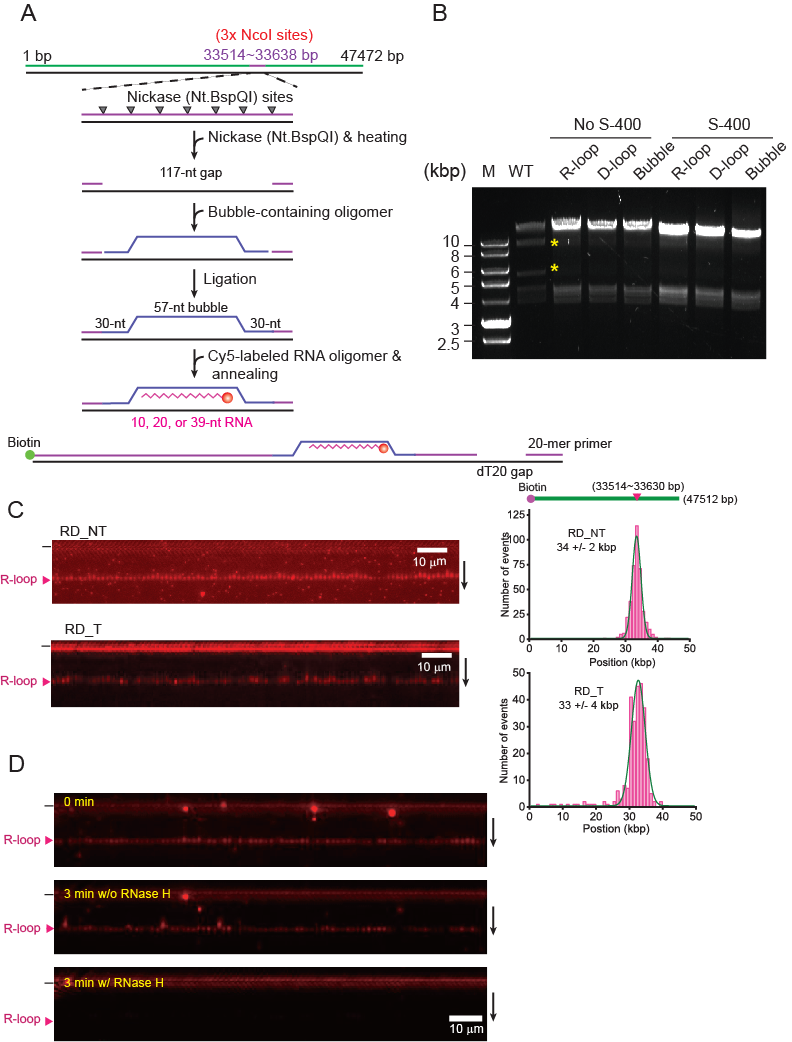


A. Schematic procedure to prepare λ-DNA containing a single R-loop. Engineered λ-DNA (λ-I3) is used, which has seven nickase sites between 33,514 bp and 33,630 bp and triple NcoI sites. At the bottom, the final λ-DNA construct containing an R-loop and a primer is displayed. Cy5 is labeled at 3’ end of RNA to avoid unwanted replication from the RNA.

B. Agarose gel electrophoresis after NcoI digestion. When an R-loop, a D-loop, or a bubble structure is properly inserted, 9,611 bp and 5,661 bp fragments (yellow asterisks) are not produced because NcoI does not cleave the R-loop construct. S-400 purification to remove short oligomer fragments after nickase treatment and bubble ligation did not change the formation of R-loop and D-loop.

C. (Left) DNA curtain images for the R-loop containing λ-DNA for RD_NT (top) and RD_T (bottom). The black bar and magenta triangle at the left represent the barrier and R-loop position, respectively. The black arrow at the right represents the flow direction. (Right) The position distribution of Cy5-labeled RNA. For both RD_NT (top) and RD_T (bottom), Cy5-labeled RNA is well placed at the R-loop location. The peak was fitted by a single Gaussian function (green). The fitted peak center is 34 ± 2 kbp and 33 ± 4 kbp for RD_NT and RD_T, respectively.

D. R-loop degradation by RNase H treatment. DNA curtain images for Cy5-labeled RNA (top) before RNase H treatment (0 min), (middle) after 3 min without RNase H treatment, and (bottom) after 3 min with RNase H treatment. The black bar and magenta triangle at the left represent the barrier and R-loop position, respectively. The black arrow at the right represents the flow direction.

**Figure S4. Collision of Phi29 DNAp with RPA-bound bubble, R-loop, or D-loop**


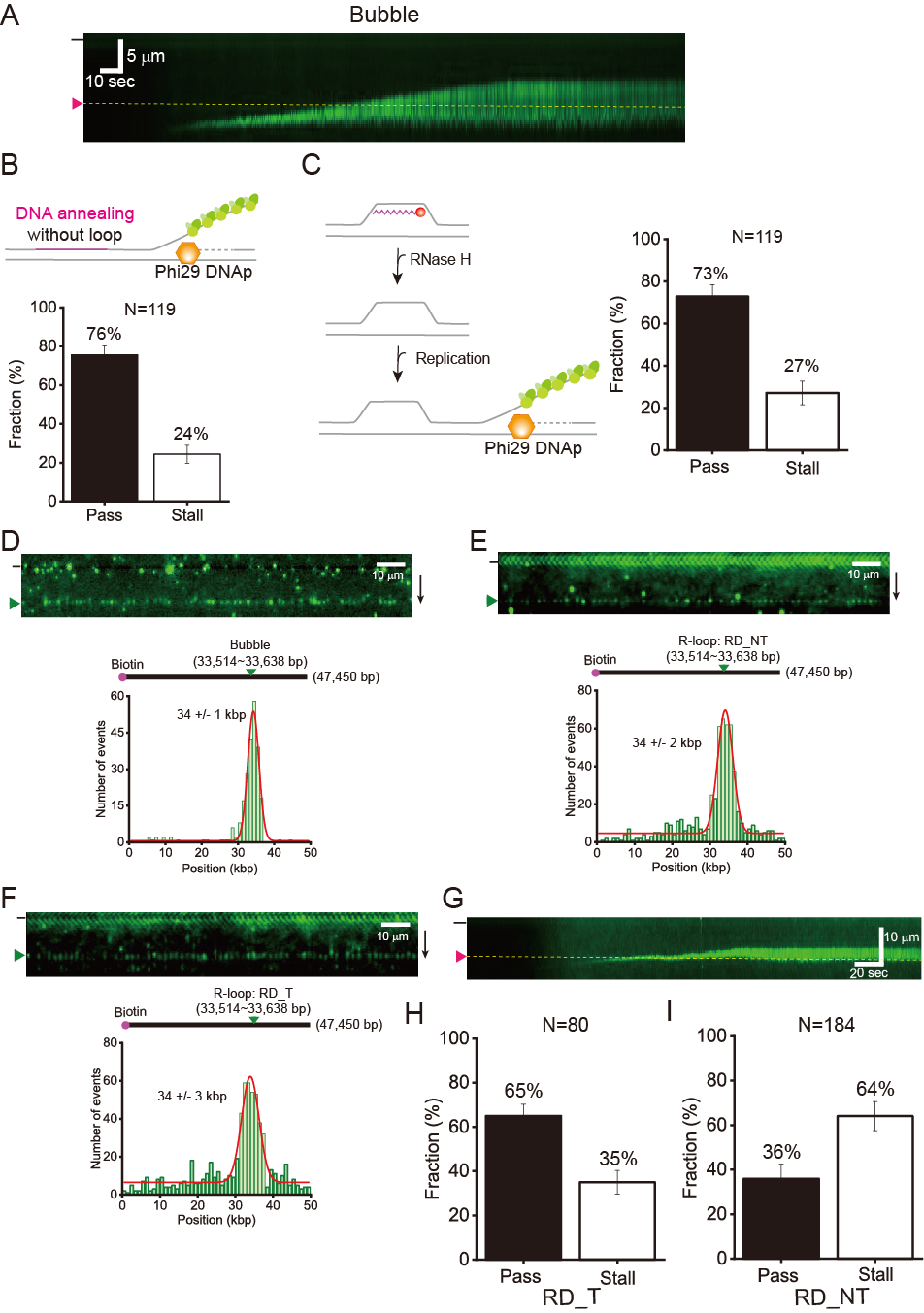


A. Kymograph for the collision of Phi29 DNAp with a bubble construct. The magenta triangle and yellow dashed line represent the bubble position, respectively. The black arrow at the right represents the flow direction.

B. (Top) Schematic of Phi29 DNAp replication through DNA duplex, which was formed by hybridization of complementary oligomer to the gap on λ-I3. (Bottom) Pass and stall fractions for duplex hybridized to the gap on λ-I3. Total number of molecules analyzed (N) is 119. Error bars represent standard deviation in triplicate.

C. Pass and stall fractions for a bubble construct, which is produced by RNase H treatment to the R-loop in λ-I3 before DNA curtain assay. (Left) Schematic of experiment. (Right) Stall and pass fractions. Total number of molecules analyzed (N) is 181. Error bars represent standard deviation in triplicate.

D. (Top) DNA curtain image for RPA-eGFP coated bubble. The black bar and green triangle at the left represent the barrier and bubble position, respectively. (Bottom) RPA binding position distribution. The peak is fitted by a single Gaussian function with the center at 34 ± 1 kbp.

E. (Top) DNA curtain image for RPA-eGFP that binds to an R-loop, in which RNA-DNA hybrid is formed at the nontemplate strand (RD_NT). The black bar and green triangle at the left represent the barrier and bubble position, respectively. (Bottom) RPA binding position distribution. The peak is fitted by a single Gaussian function with the center at 34 ± 2 kbp.

F. (Top) DNA curtain image for RPA-eGFP that binds to an R-loop, in which RNA-DNA hybrid is formed at the template strand (RD_T). The black bar and green triangle at the left represent the barrier and bubble position, respectively. (Bottom) RPA binding position distribution. The peak is fitted by a single Gaussian function with the center at 34 ± 3 kbp.

G. Kymograph showing a transient pause. The black bar at the left indicates the barrier position. The magenta triangle and yellow dashed line represent the R-loop location. The pause events were rare (~ 7%, 6 out of 85 molecules) and the pause time was 36 ± 20 sec.

H and I. Pass and stall fractions for RD_T (H) and RD_NT (I) at high RPA concentration (20 nM). Total number of molecules analyzed (N) for RD_T and RD_NT is 80 and 184, respectively. Error bars represent standard deviation in triplicate.

**Figure S5. The fate of 20-nt RNA at the R-loop after the collision of Phi29 DNAp with the R-loop**


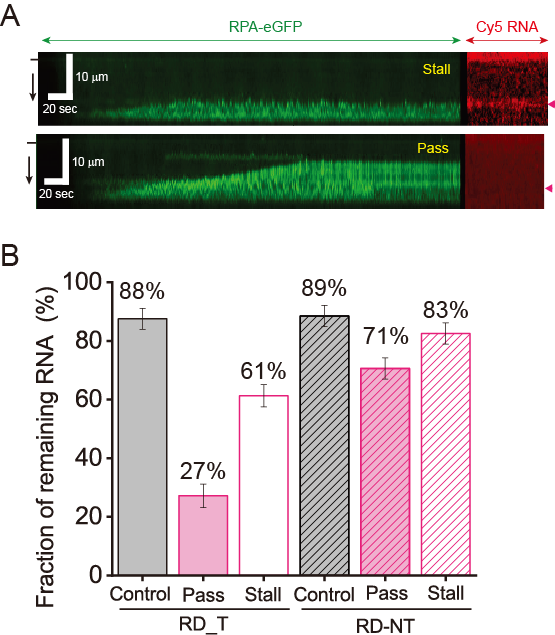


A. Kymographs showing the fate of 20-nt RNA of R-loop after the collision with Phi29 DNAp. For stalled Phi29 DNAp, RNA remains (top) whereas RNA disappears for passing Phi29 DNAp (bottom). RPA-eGFP and Cy5 imaging periods are denoted at the top of the kymographs. The black bar and black arrow at the left represent the barrier and flow direction, respectively. Magenta triangle at the right indicates the R-loop position.

B. Fractions of remaining 20-nt RNA at the R-loop after the collision with Phi29 DNAp. Control represents the fraction of remaining 20-nt RNA in the absence of Phi29 DNAp, which may result from spontaneous dissociation or photobleaching of Cy5. Total number of molecules analyzed (N) for control, pass, and stall of RD_NT is 130, 64, and 91, respectively. Total number of molecules analyzed (N) for control, pass, and stall of RD_T is 120, 81, and 62, respectively. Error bars were obtained from the standard deviation in triplicate.

**Figure S6. Pass and stall fractions depending on RNA constructs at the R-loop**


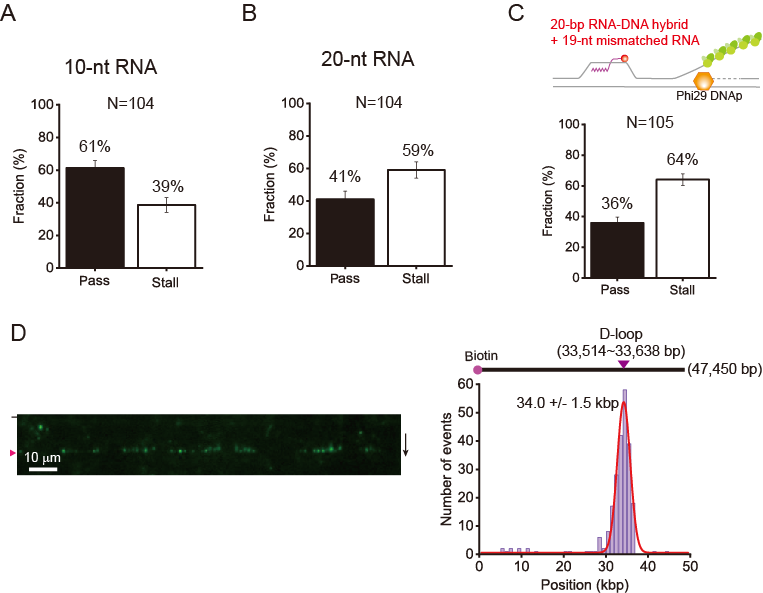


A. Pass and stall fractions for 10-nt RNA at RD_NT R-loop. Total number of molecules analyzed (N) is 104. Error bars represent standard deviation in triplicate.

B. Pass and stall fractions for 20-nt RNA at RD_NT R-loop. Total number of molecules analyzed (N) is 104. Error bars represent standard deviation in triplicate.

C. (Top) Schematic for the collision of Phi29 DNAp with the R-loop having 20-nt RNA-DNA hybrid with 19-nt 3’ overhang at the nontemplate strand. (Bottom) Pass and stall fractions for the R-loop with the 3’ overhang. Total number of molecules analyzed (N) is 105. Error bars represent standard deviation in triplicate.

D. (Top) DNA curtain image for Cy3-labeled D-loop. The black bar and magenta triangle at the left represent the barrier and D-loop position, respectively. The black arrow at the right represents the flow direction. (Bottom) Cy3-labeled DNA binding position distribution. The peak is fitted by a single Gaussian function with the center at 34.0 ± 1.5 kbp.

**Figure S7. Temperature-dependent circular dichroism for RNA-DNA hybrid and DNA duplex**


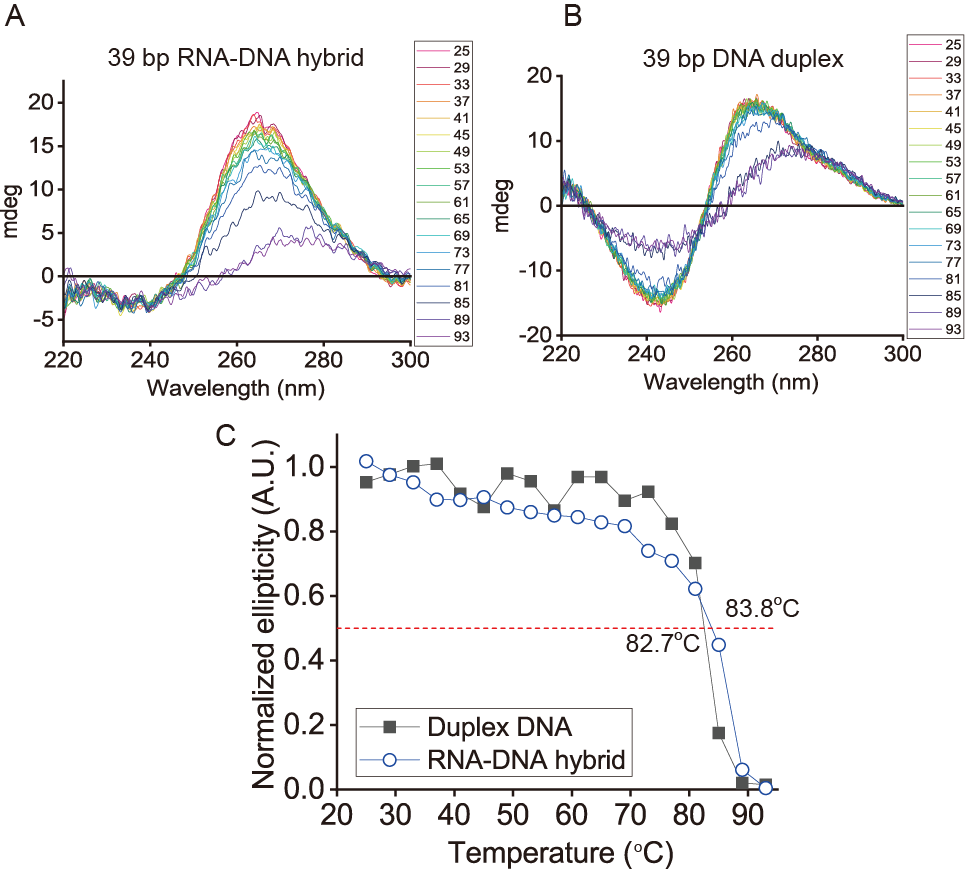


A. Circular dichroism spectra for 39 bp RNA-DNA hybrid according to temperature.

B. Circular dichroism spectra for 39 bp DNA duplex according to temperature.

C. Normalized ellipticity change at 265 nm for 39 bp DNA duplex (black square) and RNA-DNA hybrid (blue circle). The red dashed line indicates the half of the ellipticity change, indicating the melting temperature.

**Figure S8. *In vitro* G-quadruplex formation**


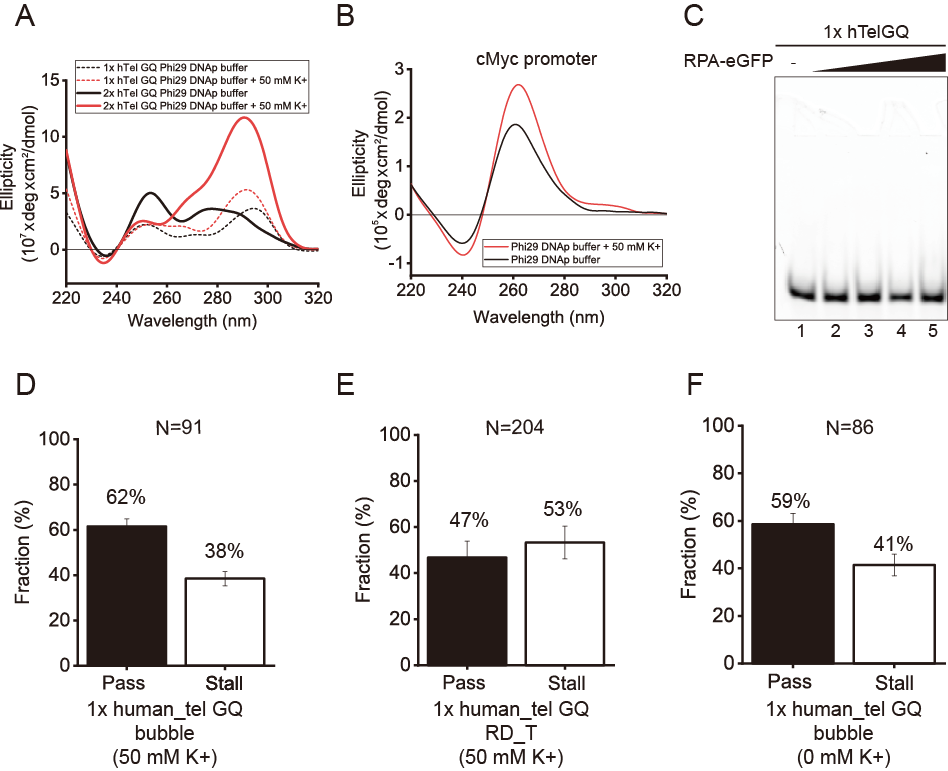


A. CD spectra for 1x hTel_GQ (d(TTAGGG)_4_) and 2x hTel_GQ (d(TTAGGG)_8_) in Phi29 DNAp buffer supplemented with or without 50 mM KCl.

B. CD spectra for 1x *cMyc* promoter GQ in Phi29 DNAp buffer supplemented with or without 50 mM KCl.

C. EMSA for RPA binding to GQ. RPA-eGFP (0, 1, 3, 10, and 30 nM) was titrated to 10 nM 1x hTel_GQ labeled with Cy5 in Phi29 DNAp buffer supplemented with 50 mM KCl.

D. Pass and stall fractions for 1x hTel_GQ in a bubble without RNA-DNA hybrid at 50 mM KCl. Total number of molecules analyzed (N) is 91. Error bars represent standard deviation in triplicate.

E. Pass and stall fractions for 1x hTel_GQ with RD_T R-loop at 50 mM KCl. Total number of molecules analyzed (N) is 204. Error bars represent standard deviation in triplicate.

F. Pass and stall fractions for 1x hTel_GQ with RD_T R-loop in the absence of KCl. Total number of molecules analyzed (N) is 86. Error bars represent standard deviation in triplicate.

**Figure S9. *In vitro* transcription of T7 RNAp**


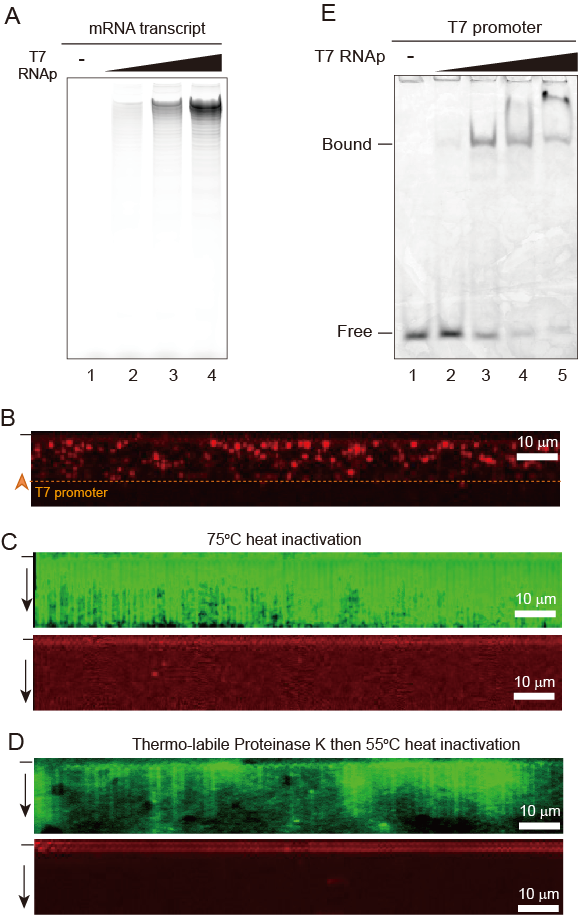


A. *In vitro* bulk transcription assay of T7 RNAp with Cy5-labeled UTP. The RNA transcripts were analyzed by 7% denaturing PAGE, Cy5 fluorescence was imaged by Typhoon RGB (Cytiva).

B. DNA curtain image for Cy5-labeled RNA transcripts that were generated by T7 RNAp. Red puncta represent individual RNA transcripts. The black bar at the left indicates the barrier position. The orange dashed line and arrow head represent T7 promoter position and its orientation.

C. DNA curtain images for YOYO-1 stained λ-DNA (top) and Cy5-labeled RNA transcripts (bottom) after the removal of T7 RNAp by 75°C heat inactivation based on the manufacturer’s protocol. RNA transcripts are removed when T7 RNAp is inactivated.

D. DNA curtain images for YOYO-1 stained DNA (top) and Cy5-labeled RNA transcripts (bottom) after the removal of T7 RNAp by thermo-labile proteinase K treatment followed by heat inactivation at 55°C. RNA transcripts are also removed when T7 RNAp is degraded.

E. EMSA for T7 RNAp (0, 10, 20, 100, and 200 nM) with T7 promoter-containing DNA.

**Supplementary Table. List of oligomers (5’-3’ direction)**

| **Name** | **Sequence** | **Mfr.** |
| --- | --- | --- |
| Lambda R-biotin | [Phosphate]AGGTCGCCGCCC[biotin] | Bioneer |
| Lambda L | [Phosphate]GGGCGGCGACCTTTTTTTTTTTTTTTTTTTTTCAGCTAGATCAGCTACTGCC | Bioneer |
| Lambda L-biotin | [Phosphate]GGGCGGCGACCT[biotin] | Bioneer |
| Lambda R | [Phosphate]AGGTCGCCGCCCTTTTTTTTTTTTTTTTTTTTCAGCTAGATCAGCTACTGCC | Bioneer |
| Phi29 Primer | GGCAGTAGCTGATCTAGCTG | Bioneer |
| Bubble Oligo* | [Phosphate]TGCATGCGGCCGCTCTTCCCATGGTGCGATTTTGTGGTT**CCCATACCGTATAACCATTTGGCTGTCCAAGCTCCGGGT**GTTTGTTTTCCGCTCTTCCCATGGTGCGATCGCTCTTCG | Bioneer |
| Lambda I3  DNA-RNA  hybrid RNA | [Phosphate]UGCAUGCGGCCGCUCUUCCCAUGGUGCGAUCGCUCUUCG | Bioneer |
| Nontemplate R-loop | ACCCGGAGCUUGGACAGCCAAAUGGUUAUACGGUAUGGG[Cy5] | Bioneer |
| Template R-loop | CUUCGUGCAUGCGGCCGCUCUUCCCAUGGUGCGAUCGCU[Cy3] | Bionics |
| Nontemplate D-loop | CTTCGTGCATGCGGCCGCTCTTCCCATGGTGCGATCGCT[Cy3] | Bioneer |
| Template D-loop | CTTCGTGCATGCGGCCGCTCTTCCCATGGTGCGATCGCT[Cy3] | Bioneer |
| Nontemplate  short R-loop | GAGCUUGGACAGCCAAAUGG[Cy5] | Bionics |
| Template  Short R-loop | UGCAUGCGGCCGCUCUUCCC[Cy5] | Bioneer |
| Nontemplate  mistmatch R-loop | ACCCGGAGCUUGGACAGCCAUUUUUUUUUUUUUUUUUUU[Cy5] | Bionics |
| Nontemplate R loop 57nt 3' 18dUTP | ACCCGGAGCUUGGACAGCCAAAUGGUUAUACGGUAUGGGUUUUUUUUUUUUUUUUUU[Cy5] | IDT Technology |
| Nontemplate R loop 57nt 5' 18dUTP | UUUUUUUUUUUUUACCCGGAGCUUGGACAGCCAAAUGGUUAUACGGUAUGGG[Cy5] | IDT Technology |
| Bubble 21 bp stem hairpin | [Phosphate]TGCATGCGGCCGCTCTTCCCATGGTGCGATTTTGTCAGTAACTGTTGCAACACACATTTTTGTGTGTTGCAACAGTTACTGTGTTTTCCGCTCTTCCCATGGTGCGATCGCTCTTCG | Bioneer |
| Bubble 15 bp stem hairpin | [Phosphate]TGCATGCGGCCGCTCTTCCCATGGTGCGATTTTGTGTCATTCTGTTGCAACACACATTTTTGTGTGTTGCAACAGTTACTGTGTTTTCCGCTCTTCCCATGGTGCGATCGCTCTTCG | Bioneer |
| Bubble 10 bp stem hairpin | [Phosphate]TGCATGCGGCCGCTCTTCCCATGGTGCGATTTTGTGTCATTGACAAGCAACACACATTTTTGTGTGTTGCAACAGTTACTGTGTTTTCCGCTCTTCCCATGGTGCGATCGCTCTTCG | Bioneer |
| Bubble 5 bp stem hairpin | [Phosphate]TGCATGCGGCCGCTCTTCCCATGGTGCGATTTTGTGTCATTGACAACGTTGACACATTTTTGTGTGTTGCAACAGTTACTGTGTTTTCCGCTCTTCCCATGGTGCGATCGCTCTTCG | Bioneer |
| Bubble GQ oligo**†** | TGCATGCGGCCGCTCTTCCCATGGTGCGATTTTGTGGTTCCCATA***GGGTTAGGGTTAGGGTTAGGGTTA***GCTCCGGGTGTTTGTTTTCCGCTCTTCCCATGGTGCGATCGCTCTTCG | Bioneer |
| Bubble  2xGQ oligo**††** | TGCATGCGGCCGCTCTTCCCATGGTGCGATTTTG***GGGTTAGGGTTAGGGTTAGGGTTAGGGTTAGGGTTAGGGTTAGGGTTA***GTTTTCCGCTCTTCCCATGGTGCGATCGCTCTTCG | Bioneer |
| Bubble c-myc**‡** | TGCATGCGGCCGCTCTTCCCATGGTGCGATTTTGTGGTTCCCAT***TGGGGAGGGTGGGGAGGGTGGGGAAGG***CCGGGTGTTTGTTTTCCGCTCTTCCCATGGTGCGATCGCTCTTCG | Bioneer |
| RCR primer | [Biotin]TTTTTTTTTTTTTTTTTTTTTTTTTTTTTTGTAAAACGACGGCCAGT | Bioneer |
| [Cy5] 91nt single-  stranded DNA | [Cy5]GCCAGGGACGAGGTGAACCTGCAGGTGGGCTTTTTTTTTTTTTTTTTTTTTTTTTTTTTTTTGGTAGAATTCGGCAGCGTCATGCGACGGC | Bioneer |
| 1xhTel_GQ | GGGTTAGGGTTAGGGTTAGGGTTA | Bioneer |
| 2xhTel_GQ | GGGTTAGGGTTAGGGTTAGGGTTAGGGTTAGGGTTAGGGTTAGGGTTA | Bioneer |
| C-myc | TGGGGAGGGTGGGGAGGGTGGGGAAGG | Bioneer |
| T7 promoter_EMSA††† | [FAM]GATGCATGCTAATACGACTCACTATAGATG | Bionics |
| T7 Promoter_EMSA_comp | CATCTATAGTGAGTCGTATTAGCATGCATC | Bionics |
| CD_DNA | ACCCGGAGCTTGGACAGCCAAATGGTTATACGGTATGGG | Bioneer |
| CD_DNA_comp | CCCATACCGTATAACCATTTGGCTGTCCAAGCTCCGGGT | Bioneer |

*** Bold and underbar: binding sequence of RNA for R-loop and DNA for D-loop**

**† Bold and italic: 1x human telomeric G-quadruplex sequence**

**†† Bold and italic: 2x human telomeric G-quadruplex sequence**

**‡ Bold and italic: *cMyc* promoter sequence**

**††† underbar: T7 promoter sequence**

**IV. References**

1. Gibb, B., Silverstein, T.D., Finkelstein, I.J. and Greene, E.C. (2012) Single-stranded DNA curtains for real-time single-molecule visualization of protein-nucleic acid interactions. *Anal Chem*, **84**, 7607-7612.

2. Kang, Y., Han, Y.G., Khim, K.W., Choi, W.G., Ju, M.K., Park, K., Shin, K.J., Chae, Y.C., Choi, J.H., Kim, H. *et al.* (2023) Alteration of replication protein A binding mode on single-stranded DNA by NSMF potentiates RPA phosphorylation by ATR kinase. *Nucleic Acids Research*.

3. Milligan, J.F., Groebe, D.R., Witherell, G.W. and Uhlenbeck, O.C. (1987) Oligoribonucleotide synthesis using T7 RNA polymerase and synthetic DNA templates. *Nucleic Acids Res*, **15**, 8783-8798.

4. Noren, C.J., Anthony-Cahill, S.J., Suich, D.J., Noren, K.A., Griffith, M.C. and Schultz, P.G. (1990) In vitro suppression of an amber mutation by a chemically aminoacylated transfer RNA prepared by runoff transcription. *Nucleic Acids Res*, **18**, 83-88.

5. Kang, Y., Cheon, N.Y., Cha, J., Kim, A., Kim, H.I., Lee, L., Kim, K.O., Jo, K. and Lee, J.Y. (2020) High-throughput single-molecule imaging system using nanofabricated trenches and fluorescent DNA-binding proteins. *Biotechnol Bioeng*, **117**, 1640-1648.

6. Cheon, N.Y., Kim, H.-S., Yeo, J.-E., Schärer, O.D. and Lee, J.Y. (2019) Single-molecule visualization reveals the damage search mechanism for the human NER protein XPC-RAD23B. *Nucleic Acids Research*, **47**, 8337-8347.

7. Kang, H.J., Cheon, N.Y., Park, H., Jeong, G.W., Ye, B.J., Yoo, E.J., Lee, J.H., Hur, J.-H., Lee, E.-A., Kim, H. *et al.* (2020) TonEBP recognizes R-loops and initiates m6A RNA methylation for R-loop resolution. *Nucleic Acids Research*, **49**, 269-284.
