# Supplementary material for: Direct visualization of replication and R-loop collision using single-molecule imaging
Source: Nucleic Acids Res. 2023 Nov 22;52(1):259–73. doi: 10.1093/nar/gkad1101 (PMC10783495; doi:10.1093/nar/gkad1101)
Supplement: gkad1101_Supplemental_Files [file gkad1101_supplemental_files.zip › Supplementary Movie S1 Caption.docx]

Supplementary Movie S1 Caption

Movie for a DNA curtain visualizing real-time replication of Phi29 DNA polymerase. The growing green lines represent RPA-eGFP-bound single-stranded DNA molecules that are replaced by Phi29 DNA polymerase during its replication
